# A Computational and Experimental Study of the Regulatory Mechanisms of the Complement System

**DOI:** 10.1371/journal.pcbi.1001059

**Published:** 2011-01-20

**Authors:** Bing Liu, Jing Zhang, Pei Yi Tan, David Hsu, Anna M. Blom, Benjamin Leong, Sunil Sethi, Bow Ho, Jeak Ling Ding, P. S. Thiagarajan

**Affiliations:** 1 School of Computing, National University of Singapore, Singapore; 2 NUS Graduate School for Integrative Science and Engineering, National University of Singapore, Singapore; 3 Department of Biological Sciences, National University of Singapore, National University of Singapore, Singapore; 4 Department of Laboratory Medicine, Lund University, Malmö, Sweden; 5 Emergency Medicine Department, National University Hospital, Singapore; 6 Department of Pathology, Yong Loo Lin School of Medicine, National University of Singapore, Singapore; 7 Department of Microbiology, Yong Loo Lin School of Medicine, Singapore; 8 Singapore-MIT Alliance, National University of Singapore, Singapore; Emory University, United States of America

## Abstract

The complement system is key to innate immunity and its activation is necessary for the clearance of bacteria and apoptotic cells. However, insufficient or excessive complement activation will lead to immune-related diseases. It is so far unknown how the complement activity is up- or down- regulated and what the associated pathophysiological mechanisms are. To quantitatively understand the modulatory mechanisms of the complement system, we built a computational model involving the enhancement and suppression mechanisms that regulate complement activity. Our model consists of a large system of Ordinary Differential Equations (ODEs) accompanied by a dynamic Bayesian network as a probabilistic approximation of the ODE dynamics. Applying Bayesian inference techniques, this approximation was used to perform parameter estimation and sensitivity analysis. Our combined computational and experimental study showed that the antimicrobial response is sensitive to changes in pH and calcium levels, which determines the strength of the crosstalk between CRP and L-ficolin. Our study also revealed differential regulatory effects of C4BP. While C4BP delays but does not decrease the classical complement activation, it attenuates but does not significantly delay the lectin pathway activation. We also found that the major inhibitory role of C4BP is to facilitate the decay of C3 convertase. In summary, the present work elucidates the regulatory mechanisms of the complement system and demonstrates how the bio-pathway machinery maintains the balance between activation and inhibition. The insights we have gained could contribute to the development of therapies targeting the complement system.

## Author Summary

The complement system, which is the frontline immune defense, constitutes proteins that flow freely in the blood. It quickly detects invading microbes and alerts the host by sending signals into immune responsive cells to eliminate the hostile substances. Inadequate or excessive complement activities harm the host and may lead to immune-related diseases. Thus, it is crucial to understand how the host boosts the complement activity to protect itself and simultaneously establishes tight surveillance to attain homeostasis. Towards this goal, we developed a detailed computational model of the human complement system. To overcome the challenges resulting from the large model size, we applied probabilistic approximation and inference techniques to train the model on experimental data and explored the key network features of the model. Our model-based study highlights the importance of infection-mediated microenvironmental perturbations, which alter the pH and calcium levels. It also reveals that the inhibitor, C4BP induces differential inhibition on the classical and lectin complement pathways and acts mainly by facilitating the decay of the C3 convertase. These predictions were validated empirically. Thus, our results help to elucidate the regulatory mechanisms of the complement system and potentially contribute to the development of complement-based immunomodulation therapies.

## Introduction

The complement system is pivotal to defending against invading microorganisms. The complement proteins recognize conserved pathogen-associated molecular patterns (PAMPs) on the surface of the invading pathogens [1] to initiate the innate immunity response. The complement activity also enhances adaptive immunity [2], [3] and participates in the clearance of apoptotic cells [4] as well as damaged and altered self tissue. The complement proteins in the blood normally circulate as inactive zymogens. Upon stimulation, proteases in the system cleave the zymogens to release active fragments and initiate an amplifying cascade of further cleavages. There are three major complement activation routes: the classical, the lectin and the alternative pathways [5]. Regardless of how these pathways are initiated, the complement activity leads to proteolytic activation and deposition of the major complement proteins C4 and C3, which induces phagocytosis, and the subsequent assembly of the membrane attack complex which lyses the invading microbes. However, complement is a double-edged sword; adequate complement activation is necessary for killing the bacteria and removing the apoptotic cells, while excessive complement activation can harm the host by generating inflammation and exacerbating tissue injury. Dysregulation of the balance between complement activation and inhibition can lead to rheumatoid arthritis [6], systemic lupus erythematosus [7], Alzheimer's disease [8] and age-related macular degeneration [9]. Since the final outcome of complement related diseases may be attributable to the imbalance between activation and inhibition [10], manipulation of this balance using drugs represents an interesting therapeutic opportunity awaiting further investigation. In light of this potential, complement inhibitors such as factor H and C4b-binding protein (C4BP) are critical since they play important roles in tightly controlling the proteolytic cascade of complement and avoiding excessive activation. Therefore, a systems-level understanding of activation and inhibition, as well as the roles of inhibitors, will contribute towards the development of complement-based immunomodulation therapies.

Complement is usually initiated by the interaction of several pattern-recognition receptors with the surface of pathogens. C-reactive protein (CRP) [11] and ficolins are two initiators of the classical and lectin pathways, which boost immune responses by recognizing phosphorylcholine (PC) or N-acetylglucosamine (GlcNAc), respectively, displayed on the surface of invading bacteria [12], [13], [14]. Recently, it was discovered that under local infection-inflammation conditions as reflected by pH and calcium levels, the conformations of CRP and L-ficolin change which leads to a strong interaction between them [15]. This interaction triggers crosstalk between classical and lectin pathways and induces new amplification mechanisms, which in turn reinforces the overall antibacterial activity and bacterial clearance.

On the other hand, C4BP, a major complement inhibitor is synthesized and secreted by the liver. The estimated plasma concentration of C4BP is 260 nM under normal physiological condition [16] but its plasma level can be elevated up to four-fold during inflammation [17], [18]. Through its α-chain [19], [20], C4BP modulates complement pathways by controlling C4b-mediated reactions in multiple ways [21], [22], [23]. Further, C4BP has been proposed as a therapeutic agent for complement-related autoimmune diseases on the premise that mice models supplemented with human C4BP showed attenuation in the progression of arthritis [24]. Therefore, it is important to understand the systemic effect and the underlying inhibitory mechanism of C4BP.

With this background, we constructed a detailed computational model of the complement network consisting of a system of ordinary Differential equations (ODEs). The large model size and the many unknown kinetic rate parameters lead to significant computational challenges. Using the technique developed in [25], we approximated the ODE dynamics as a dynamic Bayesian network [26] and used it to estimate the model parameters. After constructing the model, we investigated the enhancement mechanism induced by local inflammation and its interplay with the inhibition mechanism induced by C4BP.

Our studies confirmed and further elucidated the previous experimental findings [15]. Specifically, using our model we established a detailed relationship between the antimicrobial response and the strength of the crosstalk between CRP and L-ficolin as determined by various combinations of the pH and calcium levels. We also found that C4BP prevents complement over-activation and restores homeostasis, but it achieves this in two distinct ways depending on whether the complement activity was initiated by PC or GlcNAc. Finally, the computational model suggested that the major inhibitory effect of C4BP is to potentiate the natural decay of C3 convertase (C4bC2a). These findings regarding the role of C4BP were experimentally validated.

An earlier mathematical study [27] of the complement system focused on the classical pathway. This study assumed the dynamics to be linear, which is a severe restriction. A later study by Korotaevskiy et al [28] more realistically assumed the dynamics to be non-linear. It also included the alternative pathway. The main focus was to derive quantitative conclusions regarding the lag time of the immune response as the initial concentrations of the constituent proteins were varied. Relative to [28], our model additionally includes the lectin pathway and the recently identified amplification pathways induced by the crosstalk between CRP and L-ficolin [15]. On the other hand, given our focus on the up- and down- regulation mechanisms of the complement, we do not model the alternative pathway in detail since its role is to maintain a basal level of complement activation. Instead, this basal activity and the effects of other mechanisms such as C2 bypass [29] are implicitly captured by the kinetic parameters in our model.

## Results

We start with an overview of our results before getting into details. The ODE model we constructed is based on the current knowledge of the complement system. It includes the classical and lectin pathways and the recently identified amplification pathways [15]. Thus it is a more accurate reflection of the complement system in comparison to the earlier models [27], [28]. The model consist of 42 species, 45 reactions and 85 kinetic parameters with 71 of the parameters being unknown. To deal with the large number of unknown kinetic parameters, we applied the technique developed by Liu et al [25] and derived an approximation of the ODE model as a Dynamic Bayesian Network (DBN) [26]. We then performed parameter estimation using the DBN approximation and experimentally generated test data. The resulting model was validated using published observations of bacterial killing rates [15].

We next performed sensitivity analysis of the model, which showed that the interaction between CRP and L-ficolin as well as the decay of the C3 convertase played crucial roles. Since the decay of C3 convertase is one of the regulatory targets of C4BP, which had been identified as an important regulator [22], [23], [24], we decided to investigate the role of C4BP under enhanced complement activity as a part of our study.

Next we used the model to investigate the effects of pH and calcium levels on the antibacterial response guided by previous experimental findings [15]. We computed detailed response curves of the antibacterial activity as a function of pH and calcium levels which confirmed the findings in [15] and further elucidated the enhancement of the cross talk between the classical and lectin pathway under varying local inflammation conditions.

We then turned to the inhibitory role of C4BP. We found that for PC-initiated complement activation, increasing the C4BP initial concentrations delayed the time taken to achieve the peak amplitude of the response but it did not significantly alter the magnitude of the peak response itself. On the other hand, for GlcNAc-initiated complement activity, C4BP levels affected the magnitude but not the time taken to achieve this peak response. Our subsequent experimental results agreed with these observations.

Finally, we investigated how C4BP mediates its inhibitory function. According to current understanding, there are four major mechanisms through which C4BP can inhibit complement activation. Using the model, we determined that facilitating the natural decay of C3 convertase is the most important inhibitory function of C4BP. This finding was also validated experimentally.

### Model construction

**Figure 1:**
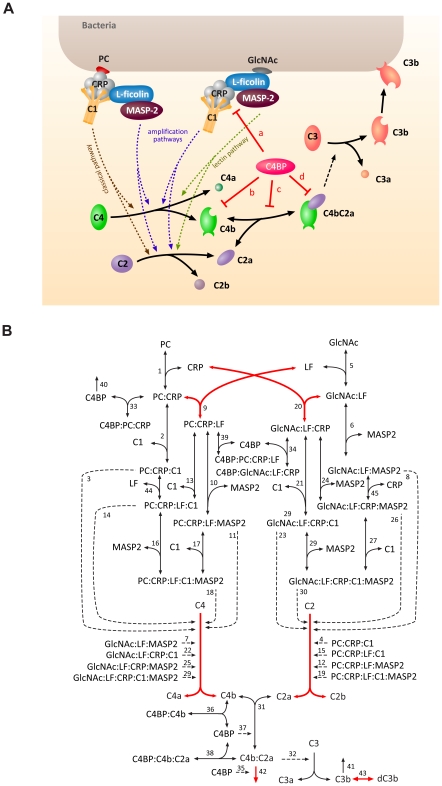
Simplified schematic representation of the complement system and reaction network diagram of the mathematical model. (A) The complement cascade is triggered when CRP or L-ficolin is recruited to the bacterial surface by binding to ligand PC (classical pathway) or GlcNAc (lectin pathway). Under inflammation condition, CRP and ficolin interact with each other and induce amplification pathways. The activated CRP and L-ficolin on the surface interacts with C1 and MASP-2 respectively and leads to the formation of the C3 convertase (C4bC2a), which cleaves C3 to C3b and C3a. Deposition of C3b initiates the opsonization, phagocytosis, and lysis. C4BP regulates the activation of complement pathways by: (a) binding to CRP, (b) accelerating the decay of the C4bC2a, (c) binding to C4b, and (d) preventing the assembly of C4bC2a (red bars). Solid arrows and dotted arrows indicate protein conversions and enzymatic reactions, respectively. (**B**) Complexes are denoted by the names of their components, separated by a “**:**”. Single-headed solid arrows characterize irreversible reactions and double-headed arrows characterize reversible reactions. Dotted arrows represent enzymatic reactions. The kinetic equations of individual reactions are presented in the supplementary material. The reactions with high global sensitivities are labeled in red.

Given our focus on the amplification and down-regulation mechanisms of complement, we included in our model only the key proteins in the classical and lectin pathways. The basal activity maintained by the alternative pathway and other mechanisms are implicitly captured by the kinetic parameters in our model. A schematic representation of the model structure is shown in **Figure 1A**. The cascade of events captured by the model can be described as follows.

The classical pathway is initiated by the binding of antibodies or CRP to antigens or PAMPs. In our model, in order to decouple the involvement of adaptive immune response, the classical pathway is triggered by the binding of CRP to PC, which is a ligand often displayed on the surface of the invading bacteria [30], [31]. Deposited CRP then binds to C1-complex (formed by C1q, two molecules of C1r, and two molecules of C1s) that is further activated. The activated C1-complex recruits C4 leading to the cleavage of C4 to its fragments, C4b and C4a. After binding of C2 to C4b, the same protease complexes are responsible for generating fragments, C2a and C2b, by cleaving C2. The C2a and C4b then form the C4bC2a complex, which is an active C3 convertase, cleaving C3 to C3a and C3b. The formation of C3b exposes a previously hidden thioester group that covalently binds to patches of hydroxyl and amino groups on the bacterial surface [32]. The surface-deposited C3b plays a central role in all subsequent steps of the complement cascade: (1) it acts as an opsonin that enhances the binding and leads to the elimination of bacteria by the phagocytes, (2) it induces the formation of membrane attack complex leading to the lysis of bacteria. Since the concentration of the deposited C3 reflects the antibacterial activity of complement, we terminated our model at this step to simplify the network.

On the other hand, the lectin pathway is initiated by the binding of mannose-binding lectin (MBL) or ficolins to PAMPs on the pathogen surface. In our model, we focused on the lectin pathway initiated by L-ficolin as it can interact with CRP and induce crosstalk between classical and lectin pathways. L-ficolin recognizes various PAMPs on the bacterial surface via the acetyl group on the GlcNAc moiety [33], [34]. Therefore, in our model the lectin pathway was triggered by binding of L-ficolin and GlcNAc onto the bacterial surface. Subsequently, a protease zymogen called MASP-2 is recruited and activated. Activated MASP-2 cleaves C4 and C2 to form C4bC2a which is C3 convertase. At this point, the classical pathway and lectin pathway merge at the cleavage step of the central complement protein, C3, and hence constitutes the endpoint of our model.

As discovered in [15], infection-induced local inflammation conditions (slight acidosis and hypocalcaemia) provoke a strong crosstalk between CRP and L-ficolin [15]. This elicits two new complement-amplification pathways, which reinforce the classical and lectin pathways. Since we aimed to study the complement activation and modulation under pathophysiological conditions, we included these two amplification pathways (**Figure 1A** **, purple**) in our model. Infection by bacteria containing PC will induce the CRP:L-ficolin mediated amplification pathway: PC→CRP:L-ficolin→MASP2→C4→C2→C3. On the other hand, infection by bacteria containing GlcNAc will induce the CRP:L-ficolin mediated amplification pathway: GlcNAc→CRP:L-ficolin→C1→C4→C2→C3.

The complement allows a rapid attack to intruding bacteria while at the same time protecting the host cells from over-activation. C4BP, a major inhibitor of complement activation, was reported to either accelerate the decay of the convertases or aid proteolytic inactivation of key players in the pathway into inactive forms such as factor H [32] but the systemic effect of C4BP has remained unclear. Hence, in our model, we included this major multifunctional inhibitor. Upstream of the complement cascade, C4BP competes with C1 for the immobilized CRP [23]. Downstream to this, C4BP binds to C4b and serves as a cofactor to the plasma serine protease factor I in the cleavage of C4b both in the fluid phase and when C4b is deposited on bacterial surfaces [21]. In addition, C4BP is able to prevent the assembly of the C3 convertase and accelerate the natural decay of the complex [35]. All of the above effects of C4BP are considered in our model and the relevant components are depicted as red bars in **Figure 1A**.

The reaction network diagram of the model is shown in **Figure 1B**. Processes such as protein association, degradation and translocation are modeled with mass action kinetics and processes such as cleavage, activation and inhibition with Michaelis-Menten kinetics.

The resulting ODE model consists of 42 species, 45 reactions and 85 kinetic parameters with 71 unknown. The details can be found in the supporting information (**Text S1**).

Due to the large model size and many unknown kinetic parameters, tasks such as parameter estimation and sensitivity analyses became very challenging. Hence, we applied the probabilistic approximation technique developed by Liu et al [25] to derive a simpler model based on the standard probabilistic graphical formalism called Dynamic Bayesian Networks (DBNs) [26].

Briefly, this approximation scheme consists of the following steps: (i) Discretize the value space of each variable and parameter into a finite set of intervals. (ii) Discretize the time domain into a finite number of discrete time points. (iii) Sample the initial states of the system according to an assumed uniform distribution over certain intervals of values of the variables and parameters. (iv) Generate a trajectory for each sampled initial state and view the resulting set of trajectories as an approximation of the dynamics defined by the ODEs system. (v) Store the generated set of trajectories compactly as a dynamic Bayesian network and use Bayesian inference techniques to perform analysis. A more detailed description of this construction can be found in the Methods section while we explain in the Discussion section how we fixed the number of trajectories to be generated and the maximum time point upto which the trajectories are to be constructed.

In the ODE model the PC-initiated and GlcNAc-initiated complement cascades are merged for convenience. By suppressing these two cascades to one at a time (by setting the corresponding expressions in the reaction equations to zero), we constructed two dynamic Bayesian networks; one for the PC-initiated complement cascade and the other for GlcNAc-initiated complement cascade. The range of each variable and parameter was discretized into 6 non-equal size intervals and 5 equal size intervals, respectively. The time points of interest were set to {0, 100, 200,…,12600} (seconds). Each of the resulting DBN approximations encoded 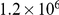 trajectories generated by sampling the initial values of the variables and the parameters from the prior, which was assumed to be uniform distributions over certain intervals. The quality of the approximations relative to the original ODEs dynamics was sufficiently high and the details can be found in the supporting information (**Figure S1**).

### Model calibration and validation

**Figure 2:**
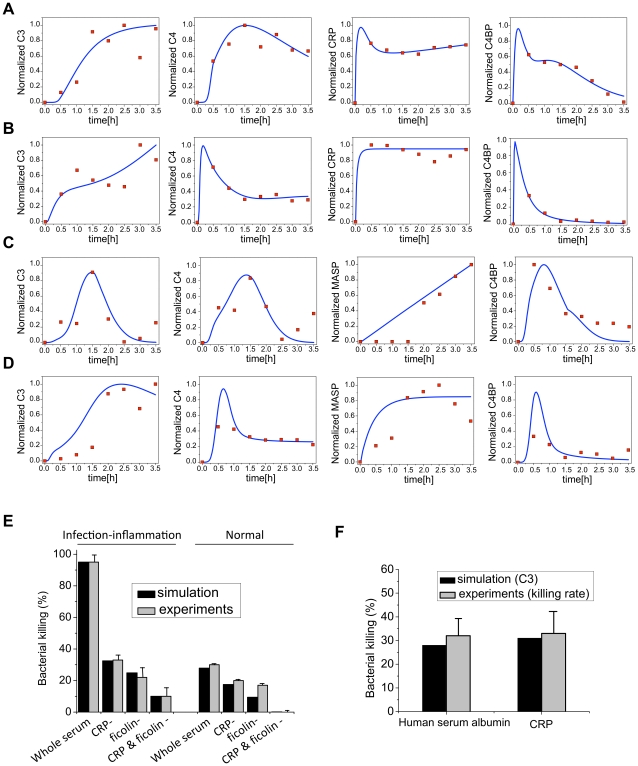
Model predictions and experimental validation. (**A–D**) Experimental and simulated dynamics of the complement pathway. The time profiles of deposited C3, C4, MASP-2, CRP and C4BP under the following four conditions are simulated using estimated parameters and compared against the experimental data: (A) PC-initiated complement activation under inflammation condition, (B) PC-initiated complement activation under normal condition. (C) GlcNAc-initiated complement activation under inflammation condition; (D) GlcNAc-initiated complement activation under normal condition. Blue solid lines depict the simulation results and red dots indicate experimental data. (E–F) Model predictions and experimental validation of effects of the crosstalk. (E) Simulation results (black bar) of end-point bacterial killing rate in whole serum, CRP depleted serum (CRP-), ficolin-depleted serum (ficolin-), both CRP- and ficolin-depleted serum (CRP- & ficolin-) under normal and infection-inflammation conditions agree with the previous experimental observations (gray bar). (F) The simulated bacterial killing effect of high CRP level agrees with the experimental data.

The values of initial concentrations and 14 kinetic parameters were obtained from literature data (**Table S1** and **Table S2**). To estimate the remaining 71 kinetic parameters, we generated test data by incubating human blood under normal and infection-inflammation conditions with beads coated with PC or GlcNAc followed by immunodetection of the deposited CRP, C4, C3 and C4BP in time series. For PC-beads, the concentration levels of deposited CRP, C4, C3 and C4BP were measured at 8 time points from 0 to 3.5 h (**Figure 2A,B** **, red dots**). For GlcNAc-beads, the concentration levels of deposited MASP-2, C4, C3 and C4BP were also measured at 8 time points from 0 to 3.5 h (**Figure 2C,D** **, red dots**).

To estimate unknown kinetic parameters, a two-stage DBN based method [25] was deployed. In the first stage, probabilistic inference applied to the discretized DBN approximation was used to find the combination of intervals of the unknown parameters that have the maximal likelihood, given the evidence consisting of the test data. As mentioned above, each unknown parameter's value space was divided into 5 equal intervals and the inference method called factored frontier algorithm [35] was used to infer the marginal distributions of the species at different time points in the DBN. We then computed the mean of each marginal distribution and compared it with the time course experimental data. To train the model by iteratively improving fitness to data, we modified the tool libSRES [36] and used its stochastic ranking evolutionary strategy (SRES), to search in the discretized parameter space consisting of 5^71^ combinations of interval values of the unknown parameters. The result of this first stage was a maximum likelihood estimate of a combination of intervals of parameter values.

In the second stage we then searched within this combination of intervals having maximal likelihood. Consequently, the size of the search space for the second stage was just 1/5^71^ of the original search space. We used the SRES search method and the parameter values thus estimated are shown in **Table S2**.

In principle, given the noisy and limited experimental data and the high dimensionality of the system, one could stop with the first stage [37] and try to work an interval of values for each parameter rather than a point value. However, in our setting we wanted to use the ODE model too for conducting *in silico* experiments such as varying initial concentrations including the down and over expression of C4BP. This would have been difficult to achieve by working solely with our current DBN approximation. We address this point again in the Discussion section.

**Figure 2A–2D** shows the comparison of the experimental time course training data (red dots) with the model simulation profiles generated using the estimated parameters (blue lines). The model predictions fit the training data well for most of the cases. In some cases, the simulations were only able to reproduce the trends of the data. This may be due to the simplifications assumed by our model and further refinement is probably necessary.

We next validated the model using previously published experimental observations [15]. In particular, normalized concentration level of deposited C3 was used to predict the antibacterial activity since C3 deposition initiated the opsonization process and the lysis of bacteria. We first simulated the concentration level of deposited C3 at 1 h under different conditions. We next normalized the results so that the maximum value among them equals to 95% which is the maximum bacterial killing rate reported in the experimental observations [15]. The normalized values were then treated as predicted bacterial killing rates. The simulation results are shown in **Figure 2E and 2F** as black bars. Consistent with the experimental data (**Figure 2E** **, grey bars**), our simulation showed that under the infection-inflammation conditions, the *P. aeruginosa*, a clinically challenging pathogen, can be efficiently killed (95% bacterial killing rate) by complement whereas under the normal condition, only 28% of the bacteria succumbed (**Figure 2E** **, black bars**). Consistent with experimental data, our simulation results show that in the patient serum, depletion of CRP or ficolin induced a significant drop in the killing rate from 95% to 33% or 25% respectively, indicating that the synergistic action of CRP and L-ficolin accounted for around 40% of the enhanced killing effect. However, in the normal serum, depletion of CRP or ficolin only resulted in a slight drop in the killing rate from 28% to 18% or 10% respectively. Furthermore, simulating a high CRP level (such as in the case of cardiovascular disease) under the normal healthy condition did not further increase the bacterial killing rate. As shown in **Figure 2F**, the simulation results matched the experimental data. Thus, our model was able to reproduce the published experimental observations shown in both **Figure 2E and 2F** with less than 10% error. This not only validated our model thus promoting its use for generating predictions, but also yielded positive evidence in support of the hypothesized amplification pathways induced by infection-inflammation condition. It also suggested that the antibacterial activity can be simulated efficiently by the level of deposited C3 and this was used to generate model predictions described in later sections.

### Sensitivity analysis

We performed local and global sensitivity analysis of the model to identify species and reactions that control complement activation during infection, and to evaluate the relative importance of initial concentrations and kinetic parameters for the model output.

**Figure 3:**
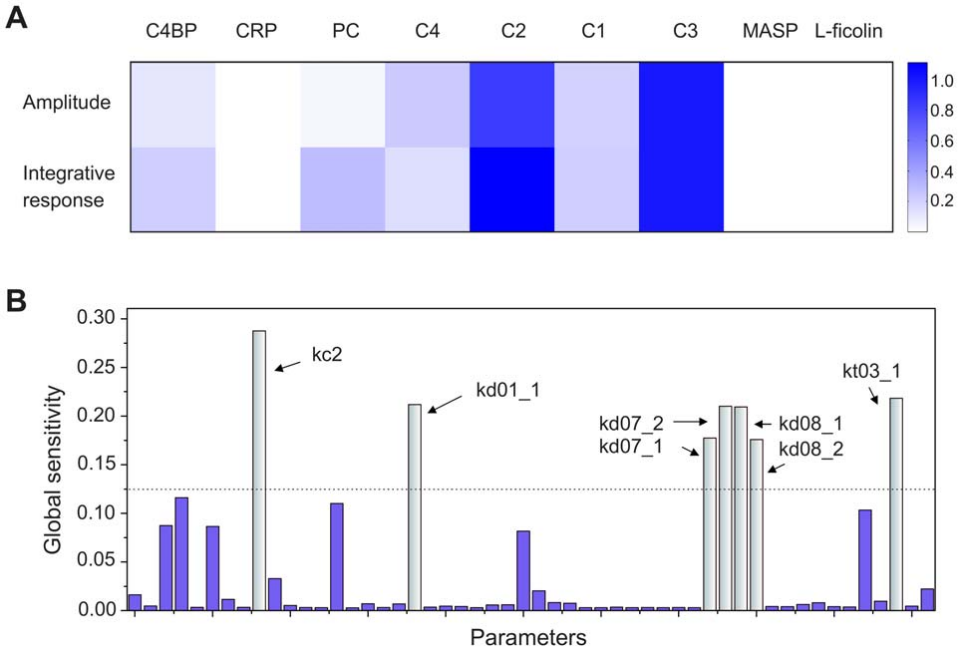
Sensitivity analysis. (A) Local sensitivities were calculated as the control coefficients of the initial protein concentrations for amplitude and integrated response of C3 deposition. (B) Global sensitivities were calculated according to the MPSA method. The most sensitive parameters are colored in light blue. *kc2* refers to the association rate of C3b with the surface. *kd01_1* refers to the association rate of CRP and ficolin. *kd07_1* and *kd_07_2* are the Michaelis-Menten constants governing the cleavage rate of C2. *kd08_1* and *kd_08_2* are the Michaelis-Menten constants governing the cleavage rate of C4. *kt03_1* refers to the decay rate of C4bC2a. Those reactions are colored in red in **Figure 1B**.

To identify critical species, we first calculated the scaled absolute local sensitivity coefficients [38] for initial concentrations of major species using the COPASI tool [39]. The model outputs were defined as the peak amplitude (maximum activation) and integrated response (area under the activation curve that reflects the overall antibacterial activity) of C3 deposition. The results are shown in **Figure 3A**. Both the peak amplitude and integrative response were strongly influenced by initial concentrations of C2 and C3, and were mildly influenced by initial concentrations of C4BP, C1 and C4. In contrast, the low sensitivities of CRP, MASP-2 and L-ficolin indicate that over-expression of these proteins is unlikely to increase the antibacterial activity. Interestingly, it was observed that the integrative response was more sensitive than the peak amplitude to the changes in the initial concentration of PC. Since the concentration of PC is correlated to the amount of invading bacteria, this result implies that the maximum complement response level may not increase as the amount of bacteria increases but the overall response (i.e. the area under the curve obtained by integrating the response level over time) will be enhanced to combat the increased number of bacteria.

In order to identify critical reactions, we next computed global sensitivities for kinetic parameters. To reduce complexity, we used the DBN approximations. Multi-parametric sensitivity analysis (MPSA) [40] was performed on the DBN for PC-initiated complement cascade (the details are presented in the Materials and Methods section). The results are shown in **Figure 3B**. Strong controls over the whole system are distributed among the parameters associated with the immobilisation of C3b with the surface, interaction between CRP and L-ficolin, cleavage of C2 and C4, and the decay of C3 convertase (see **Figure 1B**, reactions labeled in red). The sensitivity of reactions associated with C3, C2 and C4 is consistent with the local sensitivity analysis, which highlighted the significant role of major complement components. The high sensitivity of interaction of CRP and L-ficolin confirms that the overall antibacterial response depends on the strength of the crosstalk between the classical and lectin pathways. In addition, since the decay of C3 convertase is one of the regulatory targets of C4BP, the sensitivity of the system to a change in the rate of decay of C3 convertase suggested that the regulatory mechanism by C4BP plays an important role in complement. Since the critical reactions identified are common in PC- and GlcNAc-initiated complement cascades, MPSA results using the other DBN will produce similar results and hence this analysis was not performed. We next focused our investigation on the enhancement mechanism by the crosstalk and the regulatory mechanism by C4BP.

### The enhancement mechanism of the antimicrobial response

Under infection-inflammation conditions where PC-CRP:L-ficolin or GlcNAc-L-ficolin:CRP complex is formed, the amplification pathways are triggered. Model simulation showed that if C1 and L-ficolin or CRP and MASP-2 competed against each other, the antibacterial activity of the classical pathway or lectin pathway might be deprived of the amplification pathways (see **Figure S2**). Therefore, in order to achieve a stable enhancement, C1 and L-ficolin (or CRP and MASP-2) must simultaneously bind to CRP (or L-ficolin). Further, the abilities of CRP and L-ficolin to trigger subsequent complement cascade were not affected by the formation of this complex. This is consistent with the previous experimental observation that two amplification pathways co-exist with the classical and lectin pathways [15].

**Figure 4:**
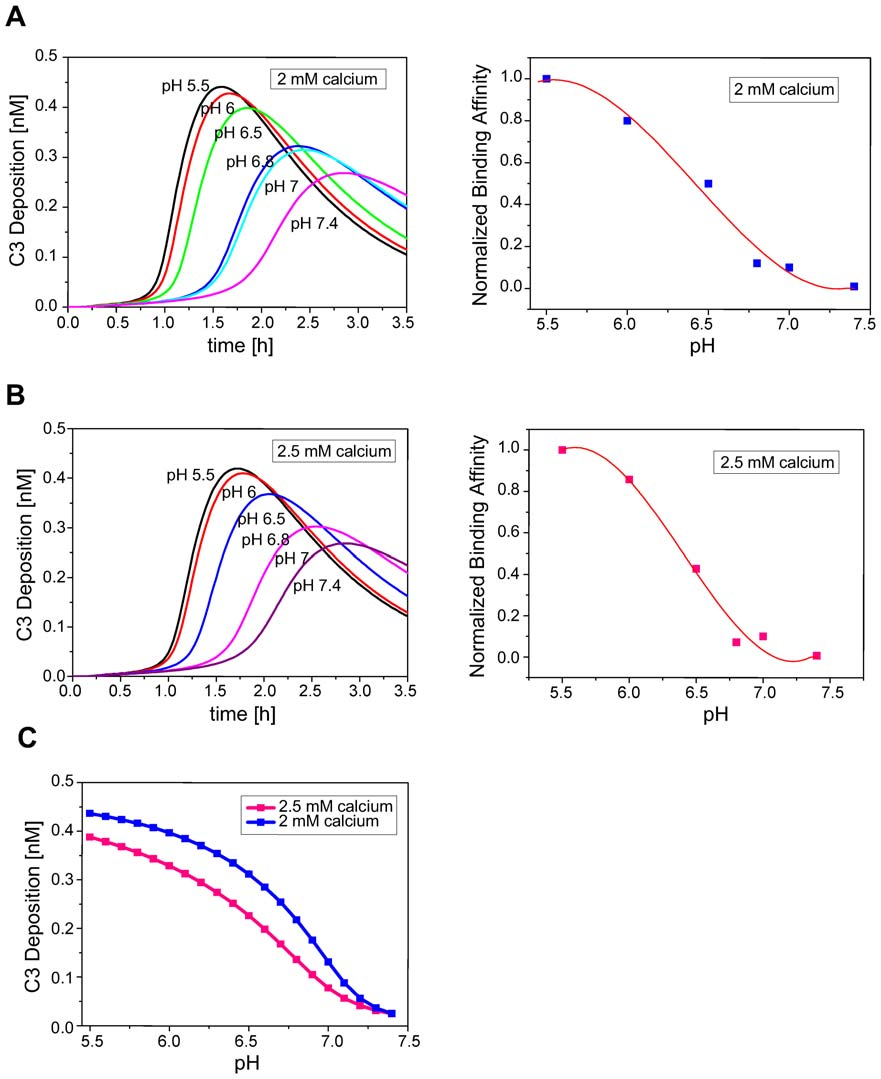
Simulation of antibacterial response with different pH and calcium level. (A) The deposited C3 time profile at pH ranging from 5.5 to 7.4, in the presence of 2 mM calcium. (B) The deposited C3 time profile at pH ranging from 5.5 to 7.4, in the presence of 2.5 mM calcium. In the right panels of (A) and (B), dots denote the normalized binding affinities of CRP and L-ficolin reported previously [15]. Curves represent the estimated functions used for predicting. (C) The pH-antibacterial response curves of complement activation in the presence of 2 mM calcium (pink) or 2.5 mM calcium (blue).

According to [15], slight acidosis and mild hypocalcaemia (pH 6.5, 2 mM calcium) prevailing at the vicinity of the infection-inflammation triggers a 100-fold stronger interaction between CRP and L-ficolin compared to the normal condition (pH 7.4, 2.5 mM calcium). This can be explained by the fact that the pH value and calcium level influence the conformations of CRP and L-ficolin which in turn govern their binding affinities. Therefore, the overall antibacterial response which is influenced by the binding affinity of CRP and L-ficolin will be sensitive to the pH value and calcium level. To confirm this and further investigate the effects of pH and calcium on the antibacterial response, we simulated the complement system under different pH and calcium conditions. Based on the previous biochemical analysis [15], we first estimated functions using polynomial regression to predict the binding affinity of CRP and L-ficolin for different pH values and calcium levels (**Figure 4A,B** **, right panel**). In the right panels of **Figure 4A and 4B**, the reported binding affinities [15] were normalized and are shown as dots. By curve fitting the dots, we estimated polynomial functions that can be used to predict the binding affinity. The curves of these functions are shown in red. We then simulated the C3 deposition dynamics using the predicted binding affinities at pH ranging from 5.5 to 7.4 in the presence of 2 mM and 2.5 mM calcium. The simulation time was chosen to be 3.5 h which is the time frame of the response peaks. The results are shown in **Figure 4A and 4B**. Under both 2 mM and 2.5 mM calcium conditions, decreasing pH induces not only the increase of the peak amplitude (maximum activation) but also hastens the peak time (time of maximum activation). To further compare the effects of the two calcium levels, the dose-response curves were generated as shown in **Figure 4C**. The antibacterial response was predicted by simulating the system for 1.5 h. At 2 mM calcium (blue curve), the antibacterial response was clearly greater than at 2.5 mM calcium (pink curve) indicating that slight hypocalcaemia enhanced the antibacterial activity in a stable manner. In addition, the pH-responses were reaching saturation levels when pH was near 5.5 (**Figure 4C**), implying that the undesirable complement-enhancement by extreme low pH condition can be avoided. This also suggests that the saturation of the pH-response was influenced by the calcium level in the milieu.

### The regulatory mechanism of C4BP on the complement system

We next investigated the complement regulation by the major inhibitor, C4BP, under infection-inflammation conditions.

**Figure 5:**
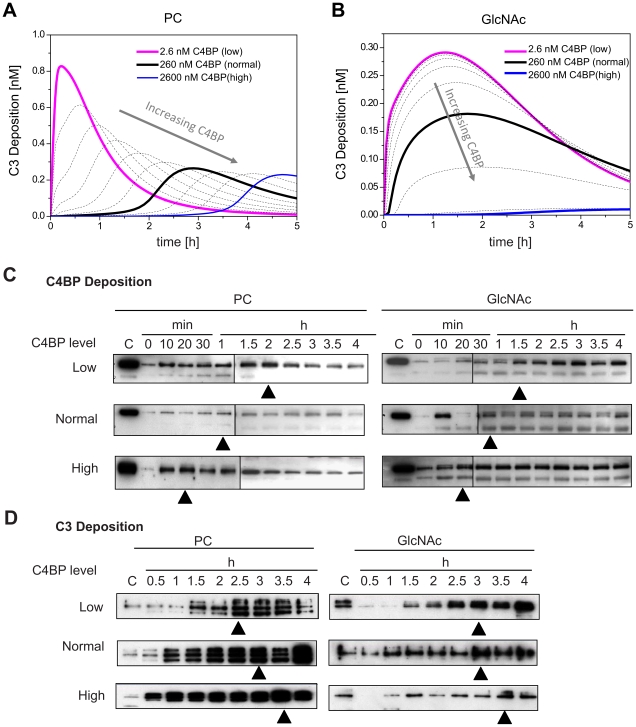
Model prediction and experimental verification of effects of C4BP under infection-inflammation condition. (A–B) Simulation results. Predicted profiles of the deposited C3 after knocking down or over-expressing C4BP in the presence of PC (A) or GlcNAc (B). (C–D) Experimental data. Profiles of deposited C4BP (C) or C3 (D) across time points of 0–4 h under infection-inflammation condition via classical pathway (triggered by PC beads) or lectin pathway (triggered by GlcNAc beads) in untreated or treated sera with increased C4BP or decreased C4BP, were studied. The deposited protein was resolved in 12% reducing SDS PAGE and detected using polyclonal sheep anti-C4BP. Same amount of pure protein was loaded to each of the gels as the positive control (labeled as “C” in the image). The black triangles point to the peaks of the time serials data.

We varied the initial concentration of C4BP and simulated the PC- and GlcNAc- initiated complement under infection-inflammation conditions. The simulation time was chosen to be 5 h which is slightly beyond the largest time point of our training experimental data. The predicted effects of the initial concentration of C4BP on the antibacterial response in terms of C3 deposition are shown in **Figure 5A and 5B**. For PC-initiated complement activation, when the starting amount of C4BP was perturbed around the normal level of 260 nM [16], increasing C4BP level only delayed the peak time but did not decrease the peak amplitude significantly. In contrast, reducing the initial C4BP level clearly hastened the complement activation and maximized the activity. Interestingly, the GlcNAc-initiated complement activation (**Figure 5B**) behaved differently from the PC-mediated complement activation (**Figure 5A**). Around the normal level of 260 nM, perturbing the initial C4BP changed the maximum activity but did not affect the peak time, suggesting that C4BP plays distinct roles in regulating the classical and lectin pathways.

To experimentally verify the model predictions, we perturbed the initial amount of C4BP in the patient sera by (i) spiking with purified C4BP (high C4BP) and (ii) reducing it by immunoprecipitation (low C4BP). The resulting C4BP levels in the normal and patient sera are shown in **Figure S3**. The sera were then incubated with PC- or GlcNAc-beads to initiate complement. Unaltered serum served as the normal control. The time profiles of the deposited C4BP level was measured over 4 h using Western blot (**Figure 5C**). Comparing the kinetic profiles in the C4BP deposition initiated by both PC and GlcNAc, we observed the following order of peak time: high C4BP>normal C4BP>low C4BP, indicating that the pre-existing initial level of C4BP was indeed the driving force controlling the deposition of complement components onto the simulated bacterial surface. We then measured the time profiles of deposited C3. **Figure 5D** shows that with PC-beads, high C4BP sera induced an early peak and low C4BP delayed the peak of C3 deposition. The peak amplitude for all three conditions was at a similar level. These observations are consistent with the simulation results shown in **Figure 5A**. With GlcNAc-beads, reducing C4BP led to a slight increase in the peak height although the peak coincided with the normal condition. In contrast, spiking the sera (high C4BP) delayed and lowered the peak amplitude of C3 deposition. Thus the experimental results broadly agree with our model predictions presented in **Figure 5B**.

**Figure 6:**
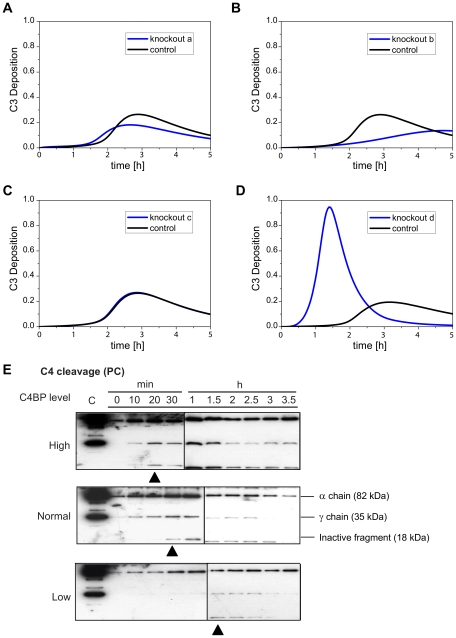
Knockout simulations reveal the major role of C4BP. (A) Simulation profiles of C3 deposition with or without reaction a. (B) Simulation profiles of C3 deposition with or without reaction b. (C) Simulation profiles of C3 deposition with or without reaction c. (D) Simulation profiles of C3 deposition with or without reaction d. Reactions (a–d) are labeled red in **Figure 1A** and explained in the caption: (a) C4BP binds to CRP, (b) C4BP binds to C4b, (c) C4BP prevents the assembly of C4bC2a, and (d) C4BP accelerates the decay of the C4bC2a,. (E) Experimental verification. Profiles of deposited cleaved/uncleaved C4 fragments across time points of 0–3.5 h under infection-inflammation condition occurring via classical pathway (triggered by PC beads) in untreated or treated sera with increased C4BP or decreased C4BP were studied. The black triangles point to the first appearance of inactive fragments.

We next investigated how C4BP mediates its inhibitory function. As shown in **Figure 1A**, the inhibitory effects of C4BP target different sites in complement: (a) binding to CRP and blocking C1, (b) preventing the formation of C4bC2a by binding to C4b, (c) acting as a cofactor for factor I in the proteolytic inactivation of C4b, and (d) accelerating the natural decay of the C4bC2a complex, which prevents the formation of C4bC2a and disrupts already formed convertase. To identify the dominant mechanism, we employed *in silico* knockout of the reactions involved for each mechanism and performed simulations. **Figure 6A–6D** shows the model predictions. Among the four inhibitory mechanisms, only the knockout of reaction (d) significantly enhanced the complement activation suggesting that facilitating the natural decay of C4bC2a (C3 convertase) is the most important inhibitory function of C4BP. This is consistent with our previous observations derived from sensitivity analysis, which identified the decay of C3 convertase as a critical reaction. In addition, as the inhibitory effect of reaction (d) is stronger than others, knocking out reaction (a) and (b) can even reduce the complement activity, which is counter-intuitive and emphasizes the significance of the systems-level understanding.

To confirm our hypothesis that the major inhibitory role of C4BP relies on accelerating the decay of C3 convertase, we measured the C4 cleavage at different time points. **Figure 6E** (black triangles) indicates the inactive C4b fragments presented from the time points of 20, 30 and 90 min under high, normal, and low C4BP conditions, suggesting that C4BP aided cleavage and inactivation of C4b, and thereby caused the natural decay of the C4bC2a.

## Discussion

Here, we developed an ODE-based dynamic model for the complement system accompanied by DBN-based approximations of the ODEs dynamics to understand how the complement activity is boosted under local inflammation conditions while a tight surveillance is established to attain homeostasis. Previously published models of complement system have focused on the classical and alternative pathways [27], [28]. Our model includes the lectin pathway and more interestingly, the recently identified amplification pathways induced by local inflammation conditions [15]. It also encompasses the regulatory effects of C4BP in the presence of enhanced complement activity.

The ODE model incorporated both the PC-initiated and GlcNAc-initiated complement together for convenience. By setting the corresponding expressions to zero one at a time, two DBN approximations were then derived; one for the PC-initiated complement cascade and the other for GlcNAc-initiated complement cascade. For constructing the DBN approximation from an ODE model, one needs to fix 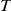, the maximal time point upto which each trajectory is to be explored and 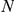, the number of trajectories to be generated.


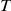
 is set to be suitably beyond the largest time point for which experimental data is available. In the present study 3.5 h, is the largest time point of our training experimental data. Based on this we set 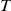 to be 5 h. After constructing the model, we simulated the system upto 10 h and found no relevant dynamics after 3.5 h.

As for the choice of 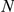, the number of trajectories, ideally one would like to specify the acceptable amount of error 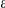 between the actual and the approximated dynamics and use 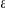 to determine 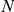. This is however difficult to achieve due to the following:

The dynamic Bayesian network we construct is a factored Markov chain. It approximates the idealized Markov chain induced by the ODEs dynamics. This idealized Markov chain is determined by the discretization of the value spaces of the variables and the parameters, the discretization of the time domain and the prior distribution of the initial states. As observed in, Liu et al [25], given an error bound, a confidence level and the transition probabilities of the idealized Markov chain, we can estimate (upper bound) the 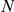 required to fall within the given error bound with the required confidence level. However, our high dimensional ODE system does not admit closed form solutions and hence the transition probabilities of the idealized Markov chain will not be computable. Hence one must make a pragmatic choice of 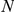.

Our approach has been to use a sampling method by which we can provide a minimum coverage of at least 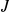 samples for each possible combination of interval values of the unknown parameters in the equation for each variable. This will ensure that the dynamics, governed by the set of equations (one for each variable) is being sufficiently sampled at least on a per equation basis. To achieve the required coverage, one will need 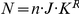 samples, where 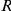 is the maximal number of unknown parameters appearing in an equation and 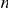 is the number of variables in the system. In our experience, 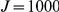 seems to be an adequate choice. Based on this, we sampled 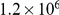 initial points and generated the corresponding trajectories. The quality of the approximations relative to the original ODEs dynamics was sufficiently high and the details can be found in the supplementary information (**Figure S1**).

How to determine 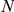 with guaranteed error bounds is however a basic problem and we are continuing to study this issue.

The study here has involved a tight integration of computational and experimental aspects. First, we used available biological information to form the biochemical network and the corresponding ODE system. We then experimentally generated test data to train the model during the process of parameter estimation. After constructing the model, one part of the computational exploration of the model was guided by the previous experimental study reported in [15]. Specifically, we computed through simulations the antibacterial response curves for varying combinations of pH values and calcium concentrations starting with the data provided in [15]. On the other hand, the second part of the study started from the computational side, namely, sensitivity analysis. Once C4BP was confirmed to be an important inhibitor through sensitivity analysis, we explored its regulatory mechanisms through simulations and generated the hypotheses concerning the differentiated influence of C4BP on PC-initiated and GlcNAc-initiated complement activity as well as the decay of the C3 convertase being the main inhibitory activity of C4BP. These hypotheses were then experimentally validated.

At present, we have used the DBN approximation to mainly aid the tasks of parameter estimation and sensitivity analysis. The key idea is to use the DBN approximation and probabilistic inference to first reduce the search space and then apply conventional search techniques to this reduced search space in the second stage. For a 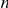-dimensional search space with 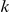 discretized intervals for each dimension, the first stage can reduce the search space by a factor of 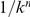.

For analyses involving multiple initial conditions (such as the *in silico* experiments involving C4BP), we found it more convenient to use the ODE model. This is due to the fact that the size of the DBN approximation increases significantly if it must encompass multiple initial conditions. Alternatively, one must construct a separate DBN for each choice of initial conditions.

Related probabilistic formalisms such as Multi Terminal Binary Decision Diagrams (MTBDDs) and Probabilistic Decision Graphs (PDGs) are also available for analysis. It is not clear at present how they can be derived directly from the ODE model. One could however try to convert our DBNs to MTBDDs for purposes of model checking [41] or develop statistical model checking methods [42].

As compact representations of the probability distributions, PDGs are, in spirit, similar to Bayesian networks [43] and can be computationally as efficient as Bayesian networks [44]. Further, probabilistic inference can be carried out with a time complexity linear in the size of the PDGs [44]. Thus, it will be an interesting future direction for us to explore the performance of PDGs in our setting.

Finally, we are aware that model construction is rarely complete. In the present setting, we included as much of the relevant and available biological information as possible in our model. Once the model was calibrated using the test data and was validated using reported bacterial killing rates, we were reasonably confident that it could be used as a platform for studying the up- and down- regulation mechanisms of the complement under local inflammation conditions. In exploring the model, we were guided by both the previous experimental study [15] and standard techniques such as sensitivity analysis. It is clear that the model will have to be refined and modified as new experimental findings become available. Indeed, we consider the systematic incremental updating of a computational model as new data become available, to be an important task [45].

Turning next to the biological insights gained from this study, we have shown that increase in PC concentration, representing the inoculum size of the invading bacteria, affected the overall classical pathway response time more than the peak amplitude.

Our model analysis confirmed that the enhancement of complement activity under infection-inflammation condition was attributable to the synergistic action of CRP and L-ficolin and supported the existence of the amplification pathways. We showed that to achieve a steady enhancement, C1 and L-ficolin (or CRP and MASP-2) should not compete with each other and the activities of CRP and L-ficolin should remain after forming the complex CRP:L-ficolin. Our computationally derived antibacterial response curves corresponding to varying pH values and calcium levels showed that the overall complement response was sensitive to pH and calcium levels.

Through model analysis we found that with under PC-activation, perturbation of the initial C4BP level only affected the peak time but not the amplitude of the response. In contrast, in the case of GlcNAc-activation, perturbation of the initial level of C4BP only affected the peak amplitude and not the peak time. These results imply that C4BP regulates the lectin pathway more stringently than the classical pathway, which is consistent with previous experimental findings [46]. Further, for PC-initiated complement cascade, the over-expression of C4BP only delays but does not “turn off” the antibacterial response. In contrast, increased C4BP can efficiently inhibit GlcNAc-initiated complement activation. This may explain previous observations that bacteria such as *Yersinia enterocolitica*, *Streptococcus pyogenes*, *Neisseria gonorrhoeae*, *Escherichia coli* K1, *Moraxella catarrhalis*, *Candida albicans*, *Bordetella pertussis* [47], [48], [49], [50], [51], [52], [53] can exploit C4BP to evade complement.

Through *in silico* knockouts, we found that, of the four documented inhibitory roles, C4BP mainly aided the natural decay of C3 convertase. As the enhancement mechanism by the crosstalk between CRP and L-ficolin occurs upstream of the cascade, we envisage C4BP acts downstream to ‘quality control’ and modulate C3 convertase activity. Thus our results suggest that efficient regulation of complement can be achieved by targeting the C3 convertase, where the complement pathways merge.

In summary, by integrating our computational model and experimental observations we have obtained novel insights into how the complement activation is enhanced during infection and how excessive complement activity may be avoided. This introduces a new level of understanding of the host defense against bacterial infection. It also provides a platform for the potential development of complement-based immunomodulation therapies by exploiting the sensitivities of the perturbations of the pH, calcium and C4BP levels.

## Materials and Methods

### Computational methods

#### Mathematical modeling

A system of ODEs was derived from the reaction network shown in **Figure 1**. The variables 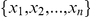 of the ODE system represent the concentration level of individual bio-molecular species. The parameters 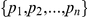 refer to the rate constants of the biochemical reactions. The rate of each reaction such as catalysis and assembly is described in terms of mass action or Michaelis-Menten laws. The rate of change of a species concentration is calculated in the usual way by summing all reaction rates that produce this species and subtracting all reaction rates that consume this species. Mathematically, the general representation of the ODE system will be a collection of equations of the form:
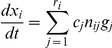
where 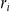 is the number of reactions associated with species 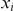 and 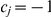 (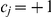) if 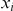 is a reactant (product) of the 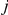 th reaction. Further, the quantities 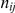 denote the stoichiometric coefficients and 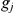 are rational functions of the form 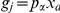 or 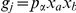 (mass action law) or 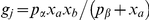 (Michaelis-Menten law) with 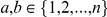 and 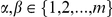, describing the reaction rates of the corresponding reactions.

The ODE model was implemented using the open source software COPASI [39]. It comprises 45 reactions, 42 species and 85 parameters. The values of initial concentrations and 14 parameters were obtained from literature data and the remaining 71 parameters were estimated. The text file of ODE equations is available in the supplementary information (**Text S1**).

#### The Dynamic Bayesian Network approximation

In order to efficiently perform computationally intensive analysis tasks, two DBN approximations [25] were constructed: one for the systems of ODEs modeling PC-initiated complement cascade and the other for the systems of ODEs modeling GlcNAc-initiated complement cascade.

Given a system of ODEs with 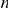 variables and 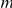 parameters as specified in the previous subsection, we assume that the dynamics is of interest only for a finite time horizon and that the states of the system are to be observed only at the discrete time points 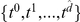. Both these assumptions are justified due to the nature of experimental data and the fact that closed form solutions will not be available and hence system states can be computed using numerical integration only for discrete time points.

Next we partition the range of each variable 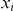 into a set of intervals 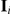 according to the assumed observation precision. We also discretize the range of each parameter 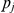 into a set of intervals, 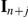. The set 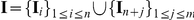 is called the *discretization*. Again, the discretization is justified by the very limited precision of experimental data. Further, given the fact that experimental data is often based on cell populations, intervals of values for the parameters are more meaningful although point values of the parameters are needed for numerical simulations. The initial values as well as the parameters of the ODE system are assumed as distributions (usually uniform) over the intervals defined by the discretization. In what follows we will loosely speak of just variables instead of distinguishing between variables and parameters.

After fixing the discretization and the distribution of initial states, we sample the initial states of the system (i.e. a vector which assigns an initial value for each variable and parameter) and generate a trajectory by numerical integration for each of the sampled initial states. The key idea is that a sufficiently large set of such trajectories is a good approximation of the dynamics defined by the ODEs system.

The second key idea is that this set of trajectories or rather, the statistical properties of these trajectories can be compactly stored in the form of a dynamic Bayesian network by exploiting the network structure of the pathway and simple counting. As a result, by querying this DBN representation using standard inferencing techniques [54] one can analyze, in a probabilistic and approximate fashion, the dynamics defined by the system of ODEs. To understand the main features of our construction, we now briefly review the notion of a DBN. For our purposes, it suffices to consider a restricted class of the so-called time variant two slice DBNs. It is a structure of the form 
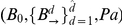
. It has 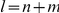 nodes at each time slice 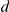. Each node represents a variable or a parameter of the ODE system at the time point 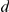 and has associated with it a random variable 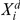, which takes as values the intervals in 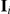. Thus 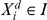 stands for the assertion “at time 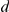, the value of the variable 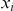 falls in the interval 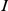. In addition, for each node one also specifies a conditional probability table which induces a probability distribution for the random variable associated with the node as explained below.


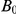
 defines the prior (initial) probability distributions 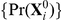 of the finite-valued random variables 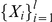. On the other hand 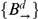 are two-slice temporal Bayesian networks for the time points 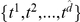. The nodes of 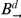 are 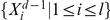 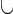 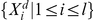 and an edge 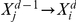 exists in 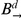 iff 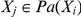, where 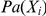 denotes the set of *parents* 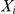. This relation is derived from the structure of the pathway as defined by the system of ODEs. More precisely, there is an edge 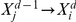 iff 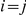 or 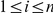 (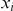 is variable) and the variable 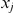 (in case 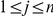) or the parameter 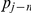 (in case 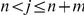) appears on the right-hand side of the equation for 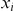. Thus, in case 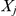 denotes a parameter, the only input edge to 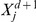 will be from 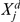, which reflects the intuition that the value of a parameter will not change once its initial value is fixed.

As mentioned earlier, each node 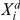 will have a condiftional probability table 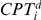 associated with it with entries of the form 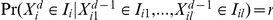, where 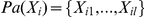 and 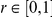. Such an entry asserts that 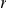 is the conditional probability of the value of the variable 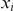 falling in the interval 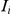 at time point 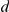, given that the value of the variable 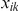 falls in the interval 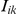 at time 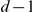 (for 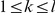). The value 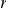 is calculated through simple counting, for instance, we sample 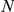 initial states and thus generate 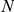 trajectories. We record, for how many of these trajectories, the value of the variable 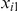 falls in the interval 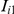 and 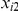 falls in the interval 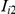 and 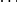 the value of 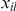 falls in the interval 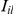 at time 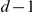. Suppose this number is 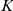, we count for how many of these 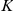 trajectories the value of the variable 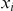 falls in the interval 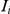 at time 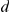. If this number is 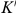 then the value 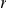 is recorded as 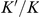.

In summary, the construction process consists of two steps: (i) derive the underlying graph of the DBN approximation by exploiting the structure of the ODEs, (ii) fill up the entries of the conditional probability tables associated with the nodes of the DBN approximation by sampling the prior distributions, performing numerical integration for each sample, discretizing generated trajectories by the predefined intervals and computing the conditional probabilities by simple counting. Our implementation of this scheme is available at our website (http://www.comp.nus.edu.sg/~rpsysbio/pada). It is a Java program that can read SBML files and generate parallelized code for construction process to be executed on clusters.

A DBN, being a special kind of Bayesian network, encodes a large dimensional joint probability distribution. Exploiting the underlying graphical structure, one can extract the joint marginal probabilities of specific combinations of variables at various time points which in turn can be built up from the one-dimensional marginal probabilities 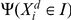. These “atomic” marginal probabilities can be computed inductively using the structure of the DBN and the entries in the conditional probability tables associated with the nodes. Clearly 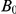 specifies these probabilities for the initial time point. Assuming that they have been computed up to the time point 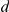, one computes 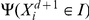 as:
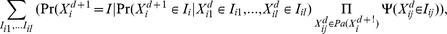
where 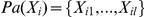. The Factored Frontier algorithm computes these marginal probabilities approximately but efficiently. It can also take into account evidence regarding initial conditions and experimental evidence presented in a suitable form.

We constructed two DBN approximations for PC-initiated classical complement pathways and GlcNAc-initiated lectin complement pathways (**Table S3** and **Table S4**). The range of each variable was discretized into 6 non-equal size intervals and the range of each parameter was discretized into 5 equal size intervals. The time points of interest were set to {0, 100, 200,…, 12600} (seconds). 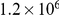 trajectories were generated up to 12600 seconds for each DBN by sampling initial states and parameters from the prior which is assumed to be uniform distributions over certain intervals (see **Table S5** and **Table S6**). The computational workload was distributed on 20 processors in a cluster and the running time was around 12 h. The quality of the approximations was evaluated by comparing the concentration profiles of selected proteins generated by the numerical simulations and the profiles generated by the DBNs using the FF algorithm. The details and the comparisons of the profiles can be found in **Figure S1**.

#### Parameter estimation

Unknown parameters were estimated using a two-stage method. A typical parameter estimation procedure involves searching in a solution space, in which each point represents a vector of parameter values. Whether a point is good or not is measured by an objective function, which captures the difference between experimental data and prediction generated by simulations using the corresponding parameters. Due to the discretized nature of the DBN approximation, the solution space is transformed into a rectilinear grid tessellated by hyperrectangles that we call *blocks*. A block dictates a combination of intervals of parameter values. Searching in this discrete space with finite number of blocks is much simpler than in the original continuous solution space. In the first stage, we adopted the stochastic ranking evolution strategy (SRES) to search for the block with minimum objective value. Further, given the fact that experimental data is often based on cell populations, intervals of values for the parameters are more meaningful although point values of the parameters are needed for numerical simulations. The SRES search was done using a modified version of the tool libSRES [36] (The modification enables one to perform search in a discrete solution space). The objective values of blocks were computed as the weighted sum of squared error between the experimental data and the expected value of marginal distributions of the variables. These marginal distributions were inferred from the DBN using the Factored Frontier algorithm [54] after supplying the chosen combination of intervals of parameter values as evidence. The first stage returned a maximum likelihood estimate of a combination of intervals of parameter values. Through probabilistic inference techniques, it can be used to carry out model analysis for fixed distribution of initial concentrations. However, we wanted to use the ODE based model too for some analysis and simulations that required perturbing the initial concentrations and a finer granularity of parameters. Hence, we further estimated the real values for unknown parameters in the second stage where we treated the resulting combination of intervals of parameter values from the first stage as the (drastically reduced) search space. In the present setting, the size of this reduced solution space is 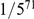 of the original one. For most parameter estimation methods, reducing the solution space increases the chance of randomly picking good starting points which in turn will lead to faster convergence. Hence the reduction of solution space we achieved using the DBN approximation contributes in this way too in improving the performance of the parameter estimation procedure.

In the second stage, we performed the standard SRES algorithm using libSRES tool to search for the point with minimum objective value. A point represents a vector of parameter values and the objective value of a point was computed as the weighted sum of squared error between the normalized experimental data and simulation profile generated using the chosen parameter values. The obtained parameter values are available in the supplementary information (**Table S2**).

#### Sensitivity analysis

Local sensitivity analysis was performed using the COPASI tool. Global sensitivity analysis was performed using a DBN-based implementation of multi-parametric sensitivity analysis (MPSA) method [40]. The MPSA procedure consists of the following steps. First, we drew a representative set of samples from the discretized parameter space. Then for each combination of intervals of parameter values, we computed the weighted sum of squared error between experimental data and the expected value of marginal distributions inferred from the DBN by supplying the selected combination of intervals of parameter values as evidence. We next classified the sampled parameter sets into two classes (good and bad) using a threshold error value and plotted the cumulative frequency of the parameter values associated with the two classes. Finally we evaluated the sensitivities as the Kolmogorov-Smirnov statistic [55] of cumulative frequency curves.

### Experimental methods

#### Antibodies, proteins & sera

Human C1 complex protein was purchased from Sigma-Aldrich (St. Louis, MO). Human C4b-binding protein was from Complement Technology (Tyler, Texas). Goat anti-rabbit secondary antibody with HRP conjugation, polyclonal rabbit anti-C3d and anti-C4c antibodies were purchased from Dako A/S (Glostrup, Denmark). Secondary anti-sheep antibody was from Upstate (Lake Placid, NY). Rabbit anti-human C4BP antibody and mouse anti-human C4BP antibody targeting N-terminal part of C4BP were raised according to standard protocols. C4BP used as standard for ELISA was purified from human plasma [56], [57]. Serum samples were obtained from healthy adults and infected patient volunteers with informed consent. As an infecion marker, the CRP levels in the serum samples were determined using the CRP Bioassay ELISA kit (BD Biosciences, San Jose, CA) to confirm the healthy and infectious status of the samples. All experiments were performed according to national and institutional guidelines on ethics and biosafety (Institutional Review Board, Reference Code: NUS-IRB 08-296).

#### Manipulation of C4BP level in the serum

The level of C4BP in the serum was increased by exogenously adding 100 µg purified C4BP protein per ml serum. The C4BP level in the serum was reduced by immunoprecipitation. One ml of serum was pre-cleared using 20 µl Protein G Sepharose (GE healthcare, Uppsala, Sweden) at 4°C for 1 h with gentle shaking. Sheep polyclonal anti-C4BP antibody (GeneTex Inc, Irvine, CA) was incubated with the pre-cleared serum with gentle shaking at 4°C for 1 h. Protein G Sepharose (20 µl) was then added to the serum containing the antibody-C4BP complex with gentle shaking at 4°C for 1 h. The supernatant with reduced C4BP level was stored. For both treated and untreated serum samples, C4BP level was measured by C4BP sandwich ELISA to ensure successful addition and depletion of C4BP (**Figure S3**). 10% (v/v) healthy serum, which was used in the subsequent experiments, was prepared by diluting the serum from healthy adult, in TBS buffer (25 mM Tris, 145 mM NaCl, pH 7.4, 2.5 mM CaCl_2_) and 10% (v/v) patient serum was prepared by diluting in MBS buffer (25 mM MES, 145 mM NaCl, pH 6.5, 2.0 mM CaCl_2_).

#### C4BP quantification by sandwich ELISA

To compare the C4BP levels between treated and untreated sera, sandwich ELISA was performed. 10 µl/ml of rabbit anti-human C4BP antibody in 50 µl coating buffer (75 mM sodium carbonate, pH 9.6) was immobilized on 96-well Maxisorp plates (Nunc, Roskilde, Denmark) by incubating overnight at 4°C. After four washes with wash buffer (50 mM Tris-HCL, pH 8.0 supplemented with 2 mM CaCl_2_, 0.15 M NaCl, 0.1% (v/v) Tween-20), the wells were blocked with blocking buffer (1% BSA (w/v) in TBS) at 37°C for 1 h. Following four washes, treated and untreated sera were diluted 2000 times in blocking buffer and 50 µl was added to the wells and incubated at 37°C for 1 h. After four washes, C4BP protein amount was detected with mouse anti-C4BP antibody (1∶15000) followed by rabbit anti-mouse HRP-conjugated secondary antibody (1∶2000). ABTS substrate (Roche Diagnostics, Mannheim, Germany) was added and the OD_405nm_ was read. Wells incubated with blocking buffer instead of serum served as a negative control.

#### Complement measurement by pull-down with GlcNAc- and PC- beads

Untreated serum or sera with increased or decreased C4BP from both healthy adults and patients were challenged with GlcNAc-Sepharose (Sigma-Aldrich) to initiate L-ficolin-mediated complement activation. 20 µl of GlcNAc beads was added to 500 µl of 10% serum. The beads were collected between 0.5 h to 4.0 h at intervals of 0.5 h. For patient's serum, the beads also underwent incubation at shorter time intervals of 0, 10 and 20 min. For CRP-mediated pathway, PC-Sepharose (Pierce, Rockford, IL) was used in place of GlcNAc-Sepharose. Beads were washed thrice with their corresponding incubation buffer and boiled in SDS-PAGE sample buffer.

#### Western blot

Protein samples of the different time points obtained from the previous step was electrophoresed on 12% SDS-PAGE. The primary antibodies used were polyclonal sheep anti-C4BP, polyclonal rabbit anti-C4c and polyclonal rabbit anti-C3d at dilutions of 1∶1000. Secondary antibodies used were rabbit anti-sheep and goat anti-rabbit at dilutions of 1∶15,000 and 1∶2000 respectively. The fractionated proteins were transferred to PVDF membranes (Bio-Rad). Membrane blots were incubated in blocking buffer (3% skimmed milk (w/v) in TBS) overnight at 4°C. Primary antibodies were diluted in TBS supplemented with 3% (w/v) BSA, 0.5% (v/v) Tween-20 and reacted with the blots with gentle shaking for 2 h at room temperature. After washing 4× for 15 min each with wash buffer (TBS supplemented with 0.5% (v/v) Tween-20), the blots were incubated with HRP conjugated secondary antibodies with gentle shaking for 2 h at room temperature. Visualization was performed with the use of SuperSignal West Pico Chemiluminescent Substrate from Thermo Scientific (Rockford, IL) and exposed through X-ray. Densitometric analysis of the blots was performed using GS-800 Calibrated Densitometer (Bio-Rad). Fixed amount of pure proteins were used as positive controls and the amounts of protein on different gels were normalized to the positive control and compared with each other. Data are representative of three independent experiments.

## Supporting Information

Figure S1 FF inference on DBN vs. ODE-based simulation. Solid lines represent nominal profiles and dash lines represent DBN inference results.
(0.09 MB PDF)

Figure S2 Simulation results of the possible outcomes of alternative models with C1 and L-ficolin or CRP and MASP-2 competition. The cross-talk may (A) does not effect, (B) up-regulate or (C) down-regulate the complement activation.
(0.20 MB TIF)

Figure S3 C4BP levels measured by C4BP sandwich ELISA for both treated and untreated serum samples.
(0.10 MB TIF)

Table S1 The initial concentrations.
(0.05 MB PDF)

Table S2 Parameter values. Known parameters are marked with *.
(0.09 MB PDF)

Table S3 DBN Structure of PC-initiated classical complement pathway.
(0.08 MB PDF)

Table S4 DBN Structure of GlcNAc-initiated classical complement pathway.
(0.08 MB PDF)

Table S5 Prior (initial) probability distribution of variables.
(0.09 MB PDF)

Table S6 Prior (initial) probability distribution of parameters.
(0.09 MB PDF)

Text S1 The ODE Model.
(0.08 MB PDF)
