# Supplementary material for: A Computational and Experimental Study of the Regulatory Mechanisms of the Complement System
Source: PLoS Comput Biol. 2011 Jan 20;7(1):e1001059. doi: 10.1371/journal.pcbi.1001059 (PMC3024260; doi:10.1371/journal.pcbi.1001059)
Supplement: Figure S1 — FF inference on DBN vs. ODE-based simulation. Solid lines represent nominal profiles and dash lines represent DBN inference results. (0.09 MB PDF) [file pcbi.1001059.s001.pdf]

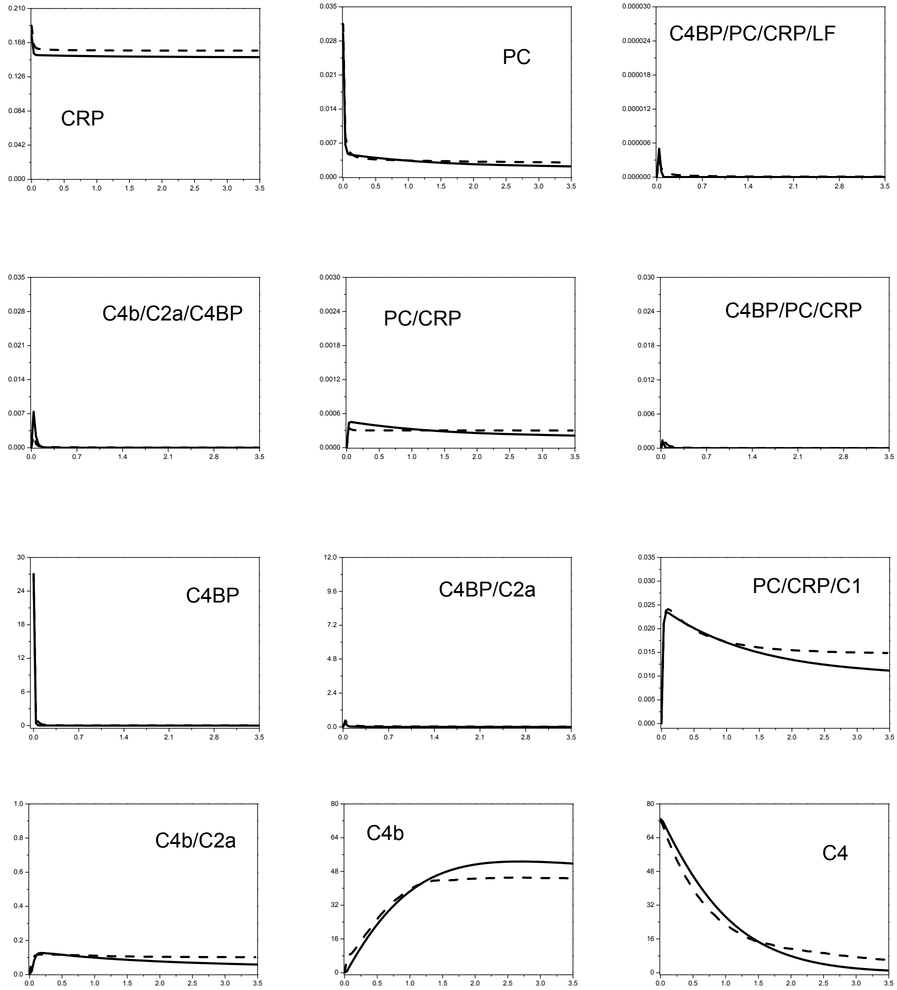

Figure S1: FF inference on DBN vs. ODE-based simulation. Solid lines represent nominal profiles and dash lines represent DBN inference results.

The quality of DBN approximations To demonstrate the quality of our DBN approximations, we implemented Monte Carlo integration for the ODE model to get good estimates by sampling and averaging. Specifically, we numerically generated 10^3 random trajectories -according to the prior- using ODEs and computed the average values of the variables at the chosen time points. The averaged trajectories projected to individual protein concentration time series values are termed to be the nominal profiles (Figure S1, solid lines). Using the FF algorithm, we inferred the marginal distributions of variables at different time points and then computed the mean of each variable over time. The resulting time profiles are shown in Figure S1 as dash line. As summarized in Figure S1, our DBN inference results was able to fit the ODE-based nominal profiles with good quality.
