# Supplementary figures and images for: A Computational and Experimental Study of the Regulatory Mechanisms of the Complement System
Source: PLoS Comput Biol. 2011 Jan 20;7(1):e1001059. doi: 10.1371/journal.pcbi.1001059 (PMC3024260; doi:10.1371/journal.pcbi.1001059)

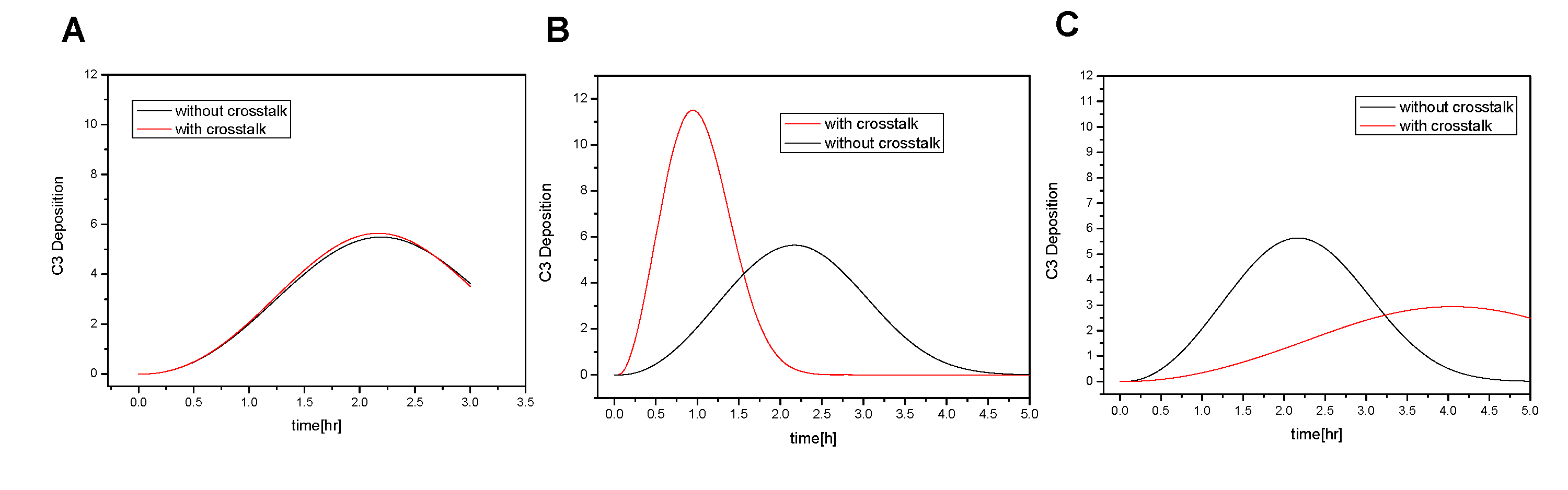

Supplement: Figure S2 — Simulation results of the possible outcomes of alternative models with C1 and L-ficolin or CRP and MASP-2 competition. The cross-talk may (A) does not effect, (B) up-regulate or (C) down-regulate the complement activation. (0.20 MB TIF) [file pcbi.1001059.s002.tif]

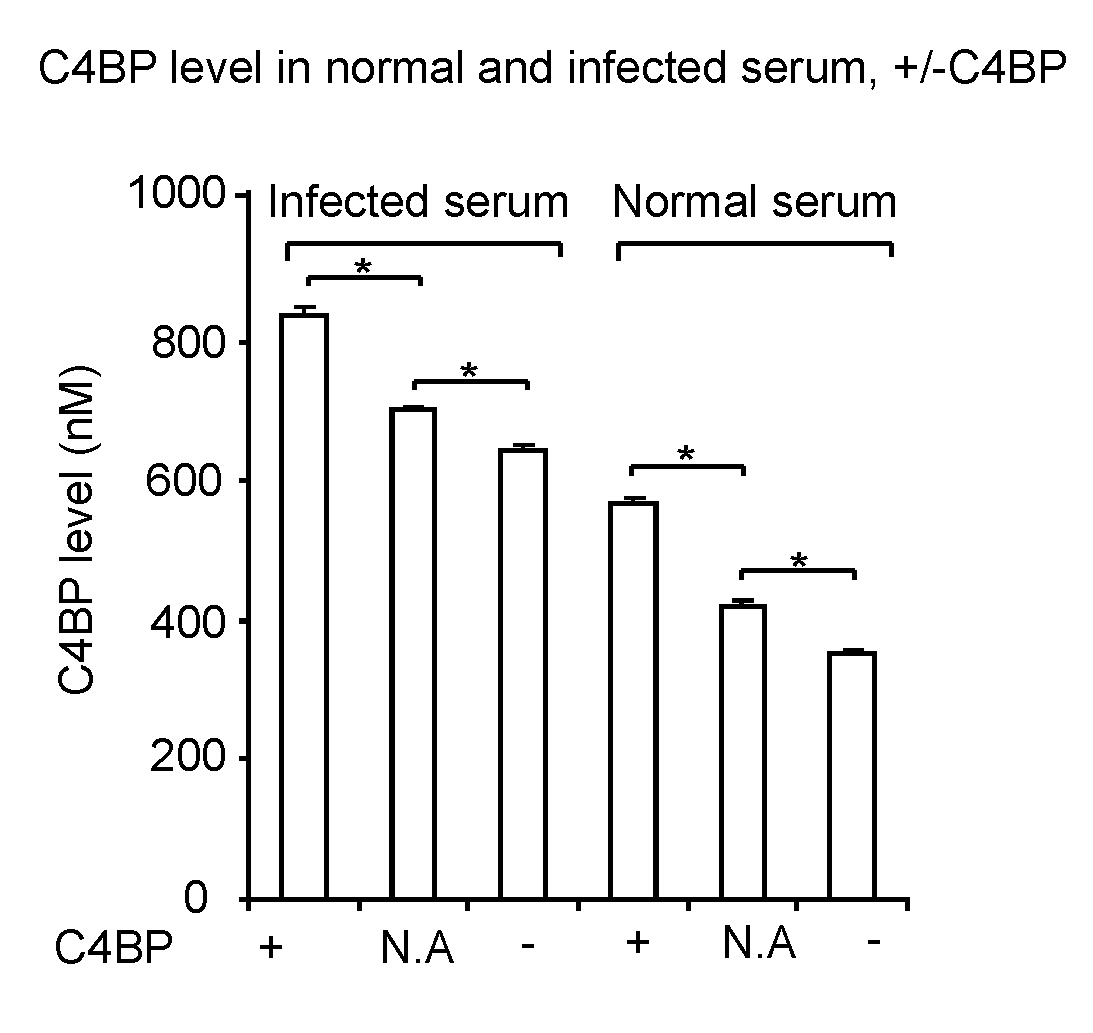

Supplement: Figure S3 — C4BP levels measured by C4BP sandwich ELISA for both treated and untreated serum samples. (0.10 MB TIF) [file pcbi.1001059.s003.tif]
