# Supplementary material for: A Computational and Experimental Study of the Regulatory Mechanisms of the Complement System
Source: PLoS Comput Biol. 2011 Jan 20;7(1):e1001059. doi: 10.1371/journal.pcbi.1001059 (PMC3024260; doi:10.1371/journal.pcbi.1001059)
Supplement: Table S1 — The initial concentrations. (0.05 MB PDF) [file pcbi.1001059.s004.pdf]

Name	Initial Concentrations [nM]
CRP	0.2
PC	0.0327796
PC/CRP	0
C4	77
C4a	0
C4b	0
C2	31
C1	247
PC/CRP/C1	0
C2a	0
C2b	0
C4b/C2a	0
C3	465
C3a	0
C3b	0
dC3b	0
MASP	0.68
dC4b/C2a	0
GlcNac	0
GlcNac/LF	0
LF	2
GlcNac/LF/MASP	0
PC/CRP/LF	0
PC/CRP/LF/MASP	0
GlcNac/LF/CRP	0
GlcNac/LF/CRP/C1	0
C4BP	26
C4BP/PC/CRP	0
C4BP/GlcNac/LF/CRP	0
iC4b/C2a	0
C4BP/C4b	0
C4b/C2a/C4BP	0
dC4b/C2a/C4BP	0
PC/CRP/LF/C1	0
C4BP/PC/CRP/LF	0
GlcNac/LF/CRP/MASP	0
PC/CRP/LF/C1/MASP	0
GlcNac/HF	0
HF	0
GlcNac/HF/MASP	0
X	0
GlcNac/LF/CRP/C1/MASP	0

Table S1: The initial concentrations.
