# Supplementary material for: A Computational and Experimental Study of the Regulatory Mechanisms of the Complement System
Source: PLoS Comput Biol. 2011 Jan 20;7(1):e1001059. doi: 10.1371/journal.pcbi.1001059 (PMC3024260; doi:10.1371/journal.pcbi.1001059)
Supplement: Table S2 — Parameter values. Known parameters are marked with *. (0.09 MB PDF) [file pcbi.1001059.s005.pdf]

| Parameter | Values |
|---|---|
| $ka01_1$ | 0.027599856 |
| $ka01_2$ | 0.0109 |
| $ka02_1$ | $7.4E-4$ |
| $ka02_2$ | 0.0011 |
| $ka03_1*$ | 2.0 |
| $ka04_1*$ | 10.5 |
| $kc01_1$ | 0.64564663 |
| $kc01_2$ | 0.19455111 |
| $kc02$ | $5.91E-4$ |
| $kc03_1$ | 0.41400447 |
| $kc03_2$ | 0.9964757 |
| $kc04_1$ | 0.97783655 |
| $ka03_2$ | 500.0 |
| $ka04_2$ | 2500.0 |
| $kd02_2$ | 0.1 |
| $kd02_1$ | 0.0368011 |
| $kd03_1$ | 66.3777 |
| $kd03_2$ | 829.116 |
| $kb01_1$ | $1.45E-4$ |
| $kb01_2$ | 0.07761722 |
| $kb02_1$ | $2.14E-7$ |
| $kb02_2$ | 0.1 |
| $kb03_1$ | 93.97925 |
| $kb03_2$ | 8815.971 |
| $kb04_1*$ | 1.1 |
| $kb04_2*$ | 2000.0 |
| $kc04_2$ | 0.19916244 |
| $kd01_1$ | $7.07E-5$ |
| $kd01_2$ | $7.23E-5$ |
| $kd04_1*$ | 1.1 |
| $kd04_2*$ | 2000.0 |
| $ke01_1$ | $7.07E-5$ |
| $ke01_2$ | $1.0E-4$ |
| $ke02_1$ | $7.4E-4$ |
| $ke02_2$ | 0.0011 |
| $ke03_1$ | 2.0 |
| $ke03_2$ | 500.0 |
| $ke04_1$ | 10.5 |
| $ke04_2$ | 2500.0 |
| $kf01_1$ | 0.9699983 |
| $kf01_2$ | 0.06902058 |
| $kf02_1$ | 0.25880134 |
| $kf02_2$ | 0.4837216 |
| $kf03$ | 0.06135372 |
| $kf04_2$ | 0.9836912 |
| $kf04_1$ | 0.6134161 |

| Parameter | Values |
|---|---|
| $kf05$ | 0.98077756 |
| $kf06_1$ | 0.613416 |
| $kf06_2$ | 0.983691 |
| $kf07_1$ | 0.613416 |
| $kf07_2$ | 0.983691 |
| $kd05_1$ | $7.4 \times 10^{-4}$ |
| $kd05_2$ | 0.0011 |
| $kd06_1*$ | 2.0 |
| $kd06_2*$ | 500.0 |
| $kd07_1*$ | 10.5 |
| $kd07_2*$ | 2500.0 |
| $ke05_1$ | $2.14 \times 10^{-7}$ |
| $ke05_2$ | 0.1 |
| $ke06_1$ | 93.97925 |
| $ke06_2$ | 8815.971 |
| $ke07_1*$ | 1.1 |
| $ke07_2*$ | 2000.0 |
| $kd08_1$ | 0.0368011 |
| $kd08_2$ | 0.1 |
| $kd09_1$ | $7.4 \times 10^{-4}$ |
| $kd09_2$ | 0.0011 |
| $kd10_1$ | 71.17058 |
| $kd10_2$ | 3796.2268 |
| $kd11_1$ | 38.96259 |
| $kd11_2$ | 5972.3066 |
| $kg01_1$ | $1.45 \times 10^{-4}$ |
| $kg01_2$ | 0.07761722 |
| $kg02_1$ | $2.14 \times 10^{-7}$ |
| $kg02_2$ | 0.1 |
| $kg03_1$ | 93.97925 |
| $kg03_2$ | 8815.971 |
| $kg04_1*$ | 1.1 |
| $kg04_2*$ | 2000.0 |
| $ke08_1$ | $2.14 \times 10^{-7}$ |
| $ke08_2$ | 0.1 |
| $ke09_1$ | $7.4 \times 10^{-4}$ |
| $ke09_2$ | 0.0011 |
| $ke10_1$ | 83.52653 |
| $ke10_2$ | 0.010678623 |
| $ke11_1$ | 79.544876 |
| $ke11_2$ | 42.56355 |
| $ktmp1$ | $3.42 \times 10^{-4}$ |
| $ktmp2$ | 0.492901 |
| $ktmp3$ | 0.0470911 |
| $ktmpf1_1$ | 0.0 |
| $ktmpf1_2$ | 0.0 |

Table S2: Parameter values. Known parameters are marked with *.
