# Supplementary material for: A Computational and Experimental Study of the Regulatory Mechanisms of the Complement System
Source: PLoS Comput Biol. 2011 Jan 20;7(1):e1001059. doi: 10.1371/journal.pcbi.1001059 (PMC3024260; doi:10.1371/journal.pcbi.1001059)
Supplement: Table S3 — DBN Structure of PC-initiated classical complement pathway. (0.08 MB PDF) [file pcbi.1001059.s006.pdf]

**Name**	**Variable**	**Parents**
CRP	$px1$	$px1$, $px2$, $px3$, $k1$, $k2$,
PC	$px2$	$px1$, $px2$, $px3$, $k1$, $k2$,
PC/CRP	$px3$	$px3$, $pt9$, $pt10$, $pt11$, $pt12$,
C4	$px4$	$px4$, $pt1$, $pt2$, $pt3$, $pt4$,
C4a	$px5$	$px4$, $px5$,
C4b	$px6$	$px6$, $pt1$, $pt3$, $pt4$, $pt34$, $pa1$,
C2	$px7$	$px7$, $pt5$, $pt6$, $pt7$, $pt8$,
C1	$px8$	$px8$, $px19$, $pt15$, $pt16$, $k66$,
PC/CRP/C1	$px9$	$px3$, $px8$, $px9$, $k3$, $k4$,
C2a	$px10$	$px10$, $pt5$, $pt13$, $pt14$, $pa2$,
C2b	$px11$	$px7$, $px11$,
C4b/C2a	$px12$	$px12$, $pt17$, $pt18$, $pt19$, $pt20$,
C3	$px13$	$px12$, $px13$, $k9$,
C3a	$px14$	$px13$, $px14$,
C3b	$px15$	$px12$, $px13$, $px15$, $k9$, $k89$,
MASP	$px16$	$px16$, $px18$, $px24$, $pt30$, $k16$, $k64$,
LF	$px17$	$px3$, $px17$, $px18$, $k28$, $k29$,
PC/CRP/LF	$px18$	$px18$, $pt21$, $pt22$, $pt23$, $pt24$,
PC/CRP/LF/MASP	$px19$	$px8$, $px19$, $pt31$, $k15$, $k66$,
C4BP	$px20$	$px20$, $pt25$, $pt26$, $pt27$, $pt28$, $k88$,
C4BP/PC/CRP	$px21$	$px3$, $px20$, $px21$, $k40$, $k41$,
C4BP/C4b	$px22$	$px6$, $px20$, $px22$, $k45$, $k46$,
C4b/C2a/C4BP	$px23$	$px12$, $px20$, $px23$, $k48$, $k49$,
PC/CRP/LF/C1	$px24$	$px16$, $px24$, $pt32$, $k53$, $k64$,
C4BP/PC/CRP/LF	$px25$	$px18$, $px20$, $px25$, $k91$, $k92$,
PC/CRP/LF/C1/MASP	$px26$	$px26$, $pt33$, $k65$, $k67$,
$TmpVar_{t1}$	$pt1$	$px4$, $px9$, $k5$, $k13$,
$TmpVar_{t2}$	$pt2$	$px4$, $px19$, $k17$, $k18$,
$TmpVar_{t3}$	$pt3$	$px4$, $px24$, $k54$, $k55$,
$TmpVar_{t4}$	$pt4$	$px4$, $px26$, $k68$, $k69$,
$TmpVar_{t5}$	$pt5$	$px7$, $px9$, $k6$, $k14$,
$TmpVar_{t6}$	$pt6$	$px7$, $px19$, $k30$, $k31$,
$TmpVar_{t7}$	$pt7$	$px7$, $px24$, $k56$, $k57$,
$TmpVar_{t8}$	$pt8$	$px7$, $px26$, $k70$, $k71$,
$TmpVar_{t9}$	$pt9$	$px1$, $px2$, $px9$, $k1$, $k4$,
$TmpVar_{t10}$	$pt10$	$px18$, $px21$, $k29$, $k41$,
$TmpVar_{t11}$	$pt11$	$px3$, $px8$, $k2$, $k3$,
$TmpVar_{t12}$	$pt12$	$px3$, $px17$, $px20$, $k28$, $k40$,
$TmpVar_{t13}$	$pt13$	$px12$, $px20$, $k8$, $k47$,
$TmpVar_{t14}$	$pt14$	$px6$, $px10$, $k7$,
$TmpVar_{t15}$	$pt15$	$px3$, $px8$, $px18$, $k3$, $k52$,
$TmpVar_{t16}$	$pt16$	$px9$, $px24$, $px26$, $k4$, $k53$, $k67$,
$TmpVar_{t17}$	$pt17$	$px2$, $px6$, $px10$, $k7$, $k49$,
$TmpVar_{t18}$	$pt18$	$px12$, $px20$, $k47$,
$TmpVar_{t19}$	$pt19$	$px12$, $px20$, $k8$, $k44$,
$TmpVar_{t20}$	$pt20$	$px12$, $px20$, $k48$, $k90$,
$TmpVar_{t21}$	$pt21$	$px16$, $px18$, $k16$, $k29$,
$TmpVar_{t22}$	$pt22$	$px3$, $px17$, $px19$, $k15$, $k28$,
$TmpVar_{t23}$	$pt23$	$px8$, $px18$, $px20$, $k52$, $k91$,
$TmpVar_{t24}$	$pt24$	$px24$, $px25$, $k53$, $k92$,
$TmpVar_{t25}$	$pt25$	$px3$, $px6$, $px20$, $k40$, $k46$,
$TmpVar_{t26}$	$pt26$	$px21$, $px22$, $k41$, $k45$,
$TmpVar_{t27}$	$pt27$	$px12$, $px18$, $px20$, $k48$, $k91$,
$TmpVar_{t28}$	$pt28$	$px23$, $px25$, $k49$, $k92$,
$TmpVar_{t29}$	$pt29$	$px12$, $px22$, $k8$, $k45$,
$TmpVar_{t30}$	$pt30$	$px19$, $px26$, $k15$, $k65$,
$TmpVar_{t31}$	$pt31$	$px16$, $px18$, $px26$, $k16$, $k67$,
$TmpVar_{t32}$	$pt32$	$px8$, $px18$, $px26$, $k52$, $k65$,
$TmpVar_{t33}$	$pt33$	$px8$, $px16$, $px19$, $px24$, $k64$, $k66$,
$TmpVar_{t34}$	$pt34$	$px6$, $px10$, $px20$, $k7$, $k46$,
$TmpVar_{a1}$	$pa1$	$pt2$, $pt18$, $pt29$,
$TmpVar_{a2}$	$pa2$	$pt6$, $pt7$, $pt8$,

Table S3: DBN Structure of PC-initiated classical complement pathway
