# Supplementary material for: A Computational and Experimental Study of the Regulatory Mechanisms of the Complement System
Source: PLoS Comput Biol. 2011 Jan 20;7(1):e1001059. doi: 10.1371/journal.pcbi.1001059 (PMC3024260; doi:10.1371/journal.pcbi.1001059)
Supplement: Table S4 — DBN Structure of GlcNAc-initiated classical complement pathway. (0.08 MB PDF) [file pcbi.1001059.s007.pdf]

**Name**	**Variable**	**Parents**
CRP	$gx1$	$gx1$, $gx15$, $gx18$, $k32$, $k33$,
C4	$gx2$	$gx2$, $gt1$, $gt2$, $gt3$, $gt4$,
C4a	$gx3$	$gx2$, $gx3$,
C4b	$gx4$	$gx4$, $gt3$, $gt4$, $gt9$, $gt10$, $ga1$,
C2	$gx5$	$gx5$, $gt5$, $gt6$, $gt7$, $gt8$,
C1	$gx6$	$gx6$, $gt11$, $gt12$,
C2a	$gx7$	$gx4$, $gx7$, $gt8$, $gt13$, $ga2$, $k7$,
C2b	$gx8$	$gx8$, $gt5$, $gt6$, $gt7$, $gt8$,
C4b/C2a	$gx9$	$gx9$, $gt14$, $gt15$, $gt16$, $k8$,
C3	$gx10$	$gx9$, $gx10$, $k9$,
C3a	$gx11$	$gx9$, $gx10$, $gx11$, $k9$,
C3b	$gx12$	$gx9$, $gx10$, $gx12$, $k9$, $k89$,
MASP	$gx13$	$gx13$, $gx15$, $gt17$, $gt18$, $k21$,
GlcNAc	$gx14$	$gx14$, $gx15$, $gx16$, $k19$, $k20$,
GlcNAc/LF	$gx15$	$gx15$, $gt19$, $gt20$, $gt21$, $k20$,
LF	$gx16$	$gx14$, $gx15$, $gx16$, $k19$, $k20$,
GlcNAc/LF/MASP	$gx17$	$gx13$, $gx15$, $gx17$, $k21$, $k22$,
GlcNAc/LF/CRP	$gx18$	$gx18$, $gt22$, $gt23$, $gt24$, $gt25$,
GlcNAc/LF/CRP/C1	$gx19$	$gx13$, $gx19$, $gt26$, $k1$, $k35$,
C4BP	$gx20$	$gx9$, $gx20$, $gt27$, $gt28$, $k48$, $k88$,
C4BP/GlcNAc/LF/CRP	$gx21$	$gx18$, $gx20$, $gx21$, $k42$, $k43$,
C4BP/C4b	$gx22$	$gx4$, $gx20$, $gx22$, $k45$, $k46$,
C4b/C2a/C4BP	$gx23$	$gx9$, $gx20$, $gx23$, $k48$, $k49$,
GlcNAc/LF/CRP/MASP	$gx24$	$gx6$, $gx24$, $gt29$, $k59$, $k66$,
GlcNAc/LF/CRP/C1/MASP	$gx25$	$gx25$, $gt30$, $k2$, $k67$,
$TmpVar_{t1}$	$gt1$	$gx2$, $gx17$, $k23$, $k24$,
$TmpVar_{t2}$	$gt2$	$gx2$, $gx19$, $k36$, $k37$,
$TmpVar_{t3}$	$gt3$	$gx2$, $gx24$, $k60$, $k61$,
$TmpVar_{t4}$	$gt4$	$gx2$, $gx25$, $k84$, $k85$,
$TmpVar_{t5}$	$gt5$	$gx5$, $gx17$, $k25$, $k26$,
$TmpVar_{t6}$	$gt6$	$gx5$, $gx19$, $k38$, $k39$,
$TmpVar_{t7}$	$gt7$	$gx5$, $gx24$, $k62$, $k63$,
$TmpVar_{t8}$	$gt8$	$gx5$, $gx25$, $k86$, $k87$,
$TmpVar_{t9}$	$gt9$	$gx9$, $gx22$, $k8$, $k45$,
$TmpVar_{t10}$	$gt10$	$gx4$, $gx7$, $gx20$, $k7$, $k46$,
$TmpVar_{t11}$	$gt11$	$gx19$, $gx25$, $k35$, $k67$,
$TmpVar_{t12}$	$gt12$	$gx6$, $gx18$, $gx24$, $k34$, $k66$,
$TmpVar_{t13}$	$gt13$	$gx9$, $gx20$, $k8$, $k47$,
$TmpVar_{t14}$	$gt14$	$gx4$, $gx7$, $gx23$, $k7$, $k49$,
$TmpVar_{t15}$	$gt15$	$gx9$, $gx20$, $k44$, $k47$,
$TmpVar_{t16}$	$gt16$	$gx9$, $gx20$, $k48$, $k90$,
$TmpVar_{t17}$	$gt17$	$gx17$, $gx24$, $gx25$, $k2$, $k22$, $k59$,
$TmpVar_{t18}$	$gt18$	$gx13$, $gx18$, $gx19$, $k1$, $k58$,
$TmpVar_{t19}$	$gt19$	$gx14$, $gx16$, $k19$,
$TmpVar_{t20}$	$gt20$	$gx17$, $gx18$, $k22$, $k33$,
$TmpVar_{t21}$	$gt21$	$gx1$, $gx13$, $gx15$, $k21$, $k32$,
$TmpVar_{t22}$	$gt22$	$gx1$, $gx15$, $gx19$, $k32$, $k35$,
$TmpVar_{t23}$	$gt23$	$gx21$, $gx24$, $k43$, $k59$,
$TmpVar_{t24}$	$gt24$	$gx13$, $gx18$, $gx20$, $k42$, $k58$,
$TmpVar_{t25}$	$gt25$	$gx6$, $gx18$, $k33$, $k34$,
$TmpVar_{t26}$	$gt26$	$gx6$, $gx18$, $gx25$, $k2$, $k34$,
$TmpVar_{t27}$	$gt27$	$gx21$, $gx22$, $gx23$, $k43$, $k45$, $k49$,
$TmpVar_{t28}$	$gt28$	$gx4$, $gx18$, $gx20$, $k42$, $k46$,
$TmpVar_{t29}$	$gt29$	$gx13$, $gx18$, $gx25$, $k58$, $k67$,
$TmpVar_{t30}$	$gt30$	$gx6$, $gx13$, $gx19$, $gx24$, $k1$, $k66$,
$TmpVar_{a1}$	$ga1$	$gx9$, $gx20$, $gt1$, $gt2$, $k47$,
$TmpVar_{a2}$	$ga2$	$gt5$, $gt6$, $gt7$,

Table S4: DBN Structure of GlcNAc-initiated classical complement pathway
