# Supplementary material for: A Computational and Experimental Study of the Regulatory Mechanisms of the Complement System
Source: PLoS Comput Biol. 2011 Jan 20;7(1):e1001059. doi: 10.1371/journal.pcbi.1001059 (PMC3024260; doi:10.1371/journal.pcbi.1001059)
Supplement: Table S5 — Prior (initial) probability distribution of variables. (0.09 MB PDF) [file pcbi.1001059.s008.pdf]

Probability distribution
$px1 \sim U(0.16808383, 0.21)$
$px2 \sim U(0.028013973, 0.035)$
$px3 \sim U(0.0, 5.988024 \times 10^{-6})$
$px4 \sim U(64.03194, 80.0)$
$px5 \sim U(0.0, 0.15968063)$
$px6 \sim U(0.0, 0.15968063)$
$px7 \sim U(28.013971, 35.0)$
$px8 \sim U(200.09981, 250.0)$
$px9 \sim U(0.0, 6.986028 \times 10^{-5})$
$px10 \sim U(0.0, 0.005988024)$
$px11 \sim U(0.0, 0.06986028)$
$px12 \sim U(0.0, 0.001996008)$
$px13 \sim U(400.19962, 500.0)$
$px14 \sim U(0.0, 0.998004)$
$px15 \sim U(0.0, 0.01996008)$
$px16 \sim U(0.5602794, 0.7)$
$px17 \sim U(1.6808383, 2.1)$
$px18 \sim U(0.0, 4.99002 \times 10^{-7})$
$px19 \sim U(0.0, 6.986028 \times 10^{-6})$
$px20 \sim U(24.011976, 30.0)$
$px21 \sim U(0.0, 5.988024 \times 10^{-5})$
$px22 \sim U(0.0, 0.023952097)$
$px23 \sim U(0.0, 6.986028 \times 10^{-5})$
$px24 \sim U(0.0, 6.986028 \times 10^{-5})$
$px25 \sim U(0.0, 5.988024 \times 10^{-8})$
$px26 \sim U(0.0, 5.988024 \times 10^{-5})$
$gx1 \sim U(0.16808383, 0.21)$
$gx2 \sim U(64.03194, 80.0)$
$gx3 \sim U(0.0, 0.15968063)$
$gx4 \sim U(0.0, 0.15968063)$
$gx5 \sim U(28.013971, 35.0)$
$gx6 \sim U(200.09981, 250.0)$
$gx7 \sim U(0.0, 9.98004 \times 10^{-4})$
$gx8 \sim U(0.0, 0.06986028)$
$gx9 \sim U(0.0, 1.9960079 \times 10^{-4})$
$gx10 \sim U(400.19962, 500.0)$
$gx11 \sim U(0.0, 0.15968063)$
$gx12 \sim U(0.0, 9.9800396 \times 10^{-5})$
$gx13 \sim U(0.5602794, 0.7)$
$gx14 \sim U(2.4011977, 3.0)$
$gx15 \sim U(0.0, 2.3952096 \times 10^{-5})$
$gx16 \sim U(1.6808383, 2.1)$
$gx17 \sim U(0.0, 3.1936127 \times 10^{-11})$
$gx18 \sim U(0.0, 1.996008 \times 10^{-8})$
$gx19 \sim U(0.0, 1.996008 \times 10^{-6})$
$gx20 \sim U(24.011976, 30.0)$
$gx21 \sim U(0.0, 2.994012 \times 10^{-7})$
$gx22 \sim U(0.0, 0.029940119)$
$gx23 \sim U(0.0, 1.1976048 \times 10^{-4})$
$gx24 \sim U(0.0, 9.98004 \times 10^{-8})$
$gx25 \sim U(0.0, 2.994012 \times 10^{-6})$

Table S5: Prior (initial) probability distribution of variables
