# Supplementary material for: A Computational and Experimental Study of the Regulatory Mechanisms of the Complement System
Source: PLoS Comput Biol. 2011 Jan 20;7(1):e1001059. doi: 10.1371/journal.pcbi.1001059 (PMC3024260; doi:10.1371/journal.pcbi.1001059)
Supplement: Table S6 — Prior (initial) probability distribution of parameters. (0.09 MB PDF) [file pcbi.1001059.s009.pdf]

Parameter	Probability distribution
$ka01_1$	$k1 \sim U(0.0, 1.0)$
$ka01_2$	$k2 \sim U(0.0, 1.0)$
$ka02_1$	$k3 \sim U(0.0, 1.0)$
$ka02_2$	$k4 \sim U(0.0, 1.0)$
$ka03_1$	$k5 \sim U(0.0, 100.0)$
$ka04_1$	$k6 \sim U(0.0, 100.0)$
$kc01_1$	$k7 \sim U(0.0, 1.0)$
$kc01_2$	$k8 \sim U(0.0, 1.0)$
$kc02$	$k9 \sim U(0.0, 1.0)$
$kc03_1$	$k10 \sim U(0.0, 1.0)$
$kc03_2$	$k11 \sim U(0.0, 1.0)$
$kc04_1$	$k12 \sim U(0.0, 1.0)$
$ka03_2$	$k13 \sim U(0.0, 10000.0)$
$ka04_2$	$k14 \sim U(0.0, 10000.0)$
$kd02_2$	$k15 \sim U(0.0, 1.0)$
$kd02_1$	$k16 \sim U(0.0, 1.0)$
$kd03_1$	$k17 \sim U(0.0, 100.0)$
$kd03_2$	$k18 \sim U(0.0, 10000.0)$
$kb01_1$	$k19 \sim U(0.0, 1.0)$
$kb01_2$	$k20 \sim U(0.0, 1.0)$
$kb02_1$	$k21 \sim U(0.0, 1.0)$
$kb02_2$	$k22 \sim U(0.0, 1.0)$
$kb03_1$	$k23 \sim U(0.0, 100.0)$
$kb03_2$	$k24 \sim U(0.0, 10000.0)$
$kb04_1$	$k25 \sim U(0.0, 100.0)$
$kb04_2$	$k26 \sim U(0.0, 10000.0)$
$kc04_2$	$k27 \sim U(0.0, 1.0)$
$kd01_1$	$k28 \sim U(0.0, 0.0707)$
$kd01_2$	$k29 \sim U(0.0, 1.0)$
$kd04_1$	$k30 \sim U(0.0, 100.0)$
$kd04_2$	$k31 \sim U(0.0, 10000.0)$
$ke01_1$	$k32 \sim U(0.0, 1.0)$
$ke01_2$	$k33 \sim U(0.0, 1.0)$
$ke02_1$	$k34 \sim U(0.0, 1.0)$
$ke02_2$	$k35 \sim U(0.0, 1.0)$
$ke03_1$	$k36 \sim U(0.0, 100.0)$
$ke03_2$	$k37 \sim U(0.0, 10000.0)$
$ke04_1$	$k38 \sim U(0.0, 100.0)$
$ke04_2$	$k39 \sim U(0.0, 10000.0)$
$kf01_1$	$k40 \sim U(0.0, 1.0)$
$kf01_2$	$k41 \sim U(0.0, 1.0)$
$kf02_1$	$k42 \sim U(0.0, 1.0)$
$kf02_2$	$k43 \sim U(0.0, 1.0)$
$kf03$	$k44 \sim U(0.0, 1.0)$
$kf04_2$	$k45 \sim U(0.0, 1.0)$
$kf04_1$	$k46 \sim U(0.0, 1.0)$

Parameter	Probability distribution
$kf05$	$k47 \sim U(0.0, 1.0)$
$kf06_1$	$k48 \sim U(0.0, 1.0)$
$kf06_2$	$k49 \sim U(0.0, 1.0)$
$kf07_1$	$k50 \sim U(0.0, 1.0)$
$kf07_2$	$k51 \sim U(0.0, 1.0)$
$kd05_1$	$k52 \sim U(0.0, 1.0)$
$kd05_2$	$k53 \sim U(0.0, 1.0)$
$kd06_1$	$k54 \sim U(0.0, 100.0)$
$kd06_2$	$k55 \sim U(0.0, 10000.0)$
$kd07_1$	$k56 \sim U(0.0, 100.0)$
$kd07_2$	$k57 \sim U(0.0, 10000.0)$
$ke05_1$	$k58 \sim U(0.0, 1.0)$
$ke05_2$	$k59 \sim U(0.0, 1.0)$
$ke06_1$	$k60 \sim U(0.0, 100.0)$
$ke06_2$	$k61 \sim U(0.0, 10000.0)$
$ke07_1$	$k62 \sim U(0.0, 100.0)$
$ke07_2$	$k63 \sim U(0.0, 10000.0)$
$kd08_1$	$k64 \sim U(0.0, 1.0)$
$kd08_2$	$k65 \sim U(0.0, 1.0)$
$kd09_1$	$k66 \sim U(0.0, 1.0)$
$kd09_2$	$k67 \sim U(0.0, 1.0)$
$kd10_1$	$k68 \sim U(0.0, 100.0)$
$kd10_2$	$k69 \sim U(0.0, 10000.0)$
$kd11_1$	$k70 \sim U(0.0, 100.0)$
$kd11_2$	$k71 \sim U(0.0, 10000.0)$
$kg01_1$	$k72 \sim U(0.0, 1.0)$
$kg01_2$	$k73 \sim U(0.0, 1.0)$
$kg02_1$	$k74 \sim U(0.0, 1.0)$
$kg02_2$	$k75 \sim U(0.0, 1.0)$
$kg03_1$	$k76 \sim U(0.0, 100.0)$
$kg03_2$	$k77 \sim U(0.0, 10000.0)$
$kg04_1$	$k78 \sim U(0.0, 100.0)$
$kg04_2$	$k79 \sim U(0.0, 10000.0)$
$ke08_1$	$k80 \sim U(0.0, 1.0)$
$ke08_2$	$k81 \sim U(0.0, 1.0)$
$ke09_1$	$k82 \sim U(0.0, 1.0)$
$ke09_2$	$k83 \sim U(0.0, 1.0)$
$ke10_1$	$k84 \sim U(0.0, 100.0)$
$ke10_2$	$k85 \sim U(0.0, 1.0)$
$ke11_1$	$k86 \sim U(0.0, 100.0)$
$ke11_2$	$k87 \sim U(0.0, 100.0)$
$ktmp1$	$k88 \sim U(0.0, 1.0)$
$ktmp2$	$k89 \sim U(0.0, 1.0)$
$ktmp3$	$k90 \sim U(0.0, 1.0)$
$ktmpf1_1$	$k91 \sim U(0.0, 1.0)$
$ktmpf1_2$	$k92 \sim U(0.0, 1.0)$

Table S6: Prior (initial) probability distribution of parameters
