# Supplementary material for: A Computational and Experimental Study of the Regulatory Mechanisms of the Complement System
Source: PLoS Comput Biol. 2011 Jan 20;7(1):e1001059. doi: 10.1371/journal.pcbi.1001059 (PMC3024260; doi:10.1371/journal.pcbi.1001059)
Supplement: Text S1 — The ODE Model. (0.08 MB PDF) [file pcbi.1001059.s010.pdf]

Text S1. The ODE Model

$$\frac{\mathrm{d}([\mathrm{CRP}])}{\mathrm{d}t} = -((\mathrm{ka01}_1 \cdot [\mathrm{PC}] \cdot [\mathrm{CRP}] - \mathrm{ka01}_2 \cdot [\mathrm{PC/CRP}])) - ((\mathrm{ke01}_1 \cdot [\mathrm{GlcNac/LF}] \cdot [\mathrm{CRP}] - \mathrm{ke01}_2 \cdot [\mathrm{GlcNac/LF/CRP}]))$$

$$\frac{\mathrm{d}([\mathrm{PC}])}{\mathrm{d}t} = -((\mathrm{ka01}_1 \cdot [\mathrm{PC}] \cdot [\mathrm{CRP}] - \mathrm{ka01}_2 \cdot [\mathrm{PC/CRP}]))$$

$$\frac{\mathrm{d}([\mathrm{PC/CRP}])}{\mathrm{d}t} = +((\mathrm{ka01}_1 \cdot [\mathrm{PC}] \cdot [\mathrm{CRP}] - \mathrm{ka01}_2 \cdot [\mathrm{PC/CRP}])) - ((\mathrm{ka02}_1 \cdot [\mathrm{PC/CRP}] \cdot [\mathrm{C1}] - \mathrm{ka02}_2 \cdot [\mathrm{PC/CRP/C1}])) - ((\mathrm{kf01}_1 \cdot [\mathrm{C4BP}] \cdot [\mathrm{PC/CRP}] - \mathrm{kf01}_2 \cdot [\mathrm{C4BP/PC/CRP}])) - ((\mathrm{kd01}_1 \cdot [\mathrm{PC/CRP}] \cdot [\mathrm{LF}] - \mathrm{kd01}_2 \cdot [\mathrm{PC/CRP/LF}]))$$

$$\frac{\mathrm{d}([\mathrm{C4}])}{\mathrm{d}t} = -\left(\frac{\mathrm{kd03}_1 \cdot [\mathrm{PC/CRP/LF/MASP}] \cdot [\mathrm{C4}]}{\mathrm{kd03}_2 + [\mathrm{C4}]}\right) - \left(\frac{\mathrm{kb03}_1 \cdot [\mathrm{GlcNac/LF/MASP}] \cdot [\mathrm{C4}]}{\mathrm{kb03}_2 + [\mathrm{C4}]}\right) - \left(\frac{\mathrm{ke03}_1 \cdot [\mathrm{GlcNac/LF/CRP/C1}] \cdot [\mathrm{C4}]}{\mathrm{ke03}_2 + [\mathrm{C4}]}\right) - \left(\frac{\mathrm{ka03}_1 \cdot [\mathrm{PC/CRP/C1}] \cdot [\mathrm{C4}]}{\mathrm{ka03}_2 + [\mathrm{C4}]}\right) - \left(\frac{\mathrm{kd06}_1 \cdot [\mathrm{PC/CRP/LF/C1}] \cdot [\mathrm{C4}]}{\mathrm{kd06}_2 + [\mathrm{C4}]}\right) - \left(\frac{\mathrm{ke06}_1 \cdot [\mathrm{GlcNac/LF/CRP/MASP}] \cdot [\mathrm{C4}]}{\mathrm{ke06}_2 + [\mathrm{C4}]}\right) - \left(\frac{\mathrm{kd10}_1 \cdot [\mathrm{PC/CRP/LF/C1/MASP}] \cdot [\mathrm{C4}]}{\mathrm{kd10}_2 + [\mathrm{C4}]}\right) - \left(\frac{\mathrm{kg03}_1 \cdot [\mathrm{GlcNac/HF/MASP}] \cdot [\mathrm{C4}]}{\mathrm{kg03}_2 + [\mathrm{C4}]}\right)$$

$$\frac{\mathrm{d}([\mathrm{C4a}])}{\mathrm{d}t} = +\left(\frac{\mathrm{kd03}_1 \cdot [\mathrm{PC/CRP/LF/MASP}] \cdot [\mathrm{C4}]}{\mathrm{kd03}_2 + [\mathrm{C4}]}\right) + \left(\frac{\mathrm{kb03}_1 \cdot [\mathrm{GlcNac/LF/MASP}] \cdot [\mathrm{C4}]}{\mathrm{kb03}_2 + [\mathrm{C4}]}\right) + \left(\frac{\mathrm{ke03}_1 \cdot [\mathrm{GlcNac/LF/CRP/C1}] \cdot [\mathrm{C4}]}{\mathrm{ke03}_2 + [\mathrm{C4}]}\right) + \left(\frac{\mathrm{ka03}_1 \cdot [\mathrm{PC/CRP/C1}] \cdot [\mathrm{C4}]}{\mathrm{ka03}_2 + [\mathrm{C4}]}\right) + \left(\frac{\mathrm{kd06}_1 \cdot [\mathrm{PC/CRP/LF/C1}] \cdot [\mathrm{C4}]}{\mathrm{kd06}_2 + [\mathrm{C4}]}\right) + \left(\frac{\mathrm{ke06}_1 \cdot [\mathrm{GlcNac/LF/CRP/MASP}] \cdot [\mathrm{C4}]}{\mathrm{ke06}_2 + [\mathrm{C4}]}\right) + \left(\frac{\mathrm{kd10}_1 \cdot [\mathrm{PC/CRP/LF/C1/MASP}] \cdot [\mathrm{C4}]}{\mathrm{kd10}_2 + [\mathrm{C4}]}\right) + \left(\frac{\mathrm{kg03}_1 \cdot [\mathrm{GlcNac/HF/MASP}] \cdot [\mathrm{C4}]}{\mathrm{kg03}_2 + [\mathrm{C4}]}\right)$$

$$\frac{\mathrm{d}([\mathrm{C4b}])}{\mathrm{d}t} = +\left(\frac{\mathrm{kd03}_1 \cdot [\mathrm{PC/CRP/LF/MASP}] \cdot [\mathrm{C4}]}{\mathrm{kd03}_2 + [\mathrm{C4}]}\right) + \left(\frac{\mathrm{kb03}_1 \cdot [\mathrm{GlcNac/LF/MASP}] \cdot [\mathrm{C4}]}{\mathrm{kb03}_2 + [\mathrm{C4}]}\right) + \left(\frac{\mathrm{ke03}_1 \cdot [\mathrm{GlcNac/LF/CRP/C1}] \cdot [\mathrm{C4}]}{\mathrm{ke03}_2 + [\mathrm{C4}]}\right) + \left(\frac{\mathrm{ka03}_1 \cdot [\mathrm{PC/CRP/C1}] \cdot [\mathrm{C4}]}{\mathrm{ka03}_2 + [\mathrm{C4}]}\right) - ((\mathrm{kf04}_1 \cdot [\mathrm{C4BP}] \cdot [\mathrm{C4b}] - \mathrm{kf04}_2 \cdot [\mathrm{C4BP/C4b}])) + (\mathrm{kf05} \cdot [\mathrm{C4b/C2a}] \cdot [\mathrm{C4BP}]) + \left(\frac{\mathrm{kd06}_1 \cdot [\mathrm{PC/CRP/LF/C1}] \cdot [\mathrm{C4}]}{\mathrm{kd06}_2 + [\mathrm{C4}]}\right) + \left(\frac{\mathrm{ke06}_1 \cdot [\mathrm{GlcNac/LF/CRP/MASP}] \cdot [\mathrm{C4}]}{\mathrm{ke06}_2 + [\mathrm{C4}]}\right) + \left(\frac{\mathrm{kd10}_1 \cdot [\mathrm{PC/CRP/LF/C1/MASP}] \cdot [\mathrm{C4}]}{\mathrm{kd10}_2 + [\mathrm{C4}]}\right) - ((\mathrm{kc01}_1 \cdot [\mathrm{C4b}] \cdot [\mathrm{C2a}] - \mathrm{kc01}_2 \cdot [\mathrm{C4b/C2a}])) + \left(\frac{\mathrm{kg03}_1 \cdot [\mathrm{GlcNac/HF/MASP}] \cdot [\mathrm{C4}]}{\mathrm{kg03}_2 + [\mathrm{C4}]}\right)$$

$$\frac{\mathrm{d}\,([\mathrm{C2}]\,)}{\mathrm{d}t} = -\left(\frac{\mathrm{kd04}_1\,\cdot\,[\mathrm{PC/CRP/LF/MASP}]\,\cdot\,[\mathrm{C2}]}{\mathrm{kd04}_2\,+\,[\mathrm{C2}]}\right) - \left(\frac{\mathrm{kb04}_1\,\cdot\,[\mathrm{GlcNac/LF/MASP}]\,\cdot\,[\mathrm{C2}]}{\mathrm{kb04}_2\,+\,[\mathrm{C2}]}\right) - \left(\frac{\mathrm{ke04}_1\,\cdot\,[\mathrm{GlcNac/LF/CRP/C1}]\,\cdot\,[\mathrm{C2}]}{\mathrm{ke04}_2\,+\,[\mathrm{C2}]}\right) - \left(\frac{\mathrm{ka04}_1\,\cdot\,[\mathrm{PC/CRP/C1}]\,\cdot\,[\mathrm{C2}]}{\mathrm{ka04}_2\,+\,[\mathrm{C2}]}\right) - \left(\frac{\mathrm{kd07}_1\,\cdot\,[\mathrm{PC/CRP/LF/C1}]\,\cdot\,[\mathrm{C2}]}{\mathrm{kd07}_2\,+\,[\mathrm{C2}]}\right) - \left(\frac{\mathrm{ke07}_1\,\cdot\,[\mathrm{GlcNac/LF/CRP/MASP}]\,\cdot\,[\mathrm{C2}]}{\mathrm{ke07}_2\,+\,[\mathrm{C2}]}\right) - \left(\frac{\mathrm{kd11}_1\,\cdot\,[\mathrm{PC/CRP/LF/C1/MASP}]\,\cdot\,[\mathrm{C2}]}{\mathrm{kd11}_2\,+\,[\mathrm{C2}]}\right) - \left(\frac{\mathrm{kg04}_1\,\cdot\,[\mathrm{GlcNac/HF/MASP}]\,\cdot\,[\mathrm{C2}]}{\mathrm{kg04}_2\,+\,[\mathrm{C2}]}\right)$$

$$\frac{\mathrm{d}\,([\mathrm{C1}]\,)}{\mathrm{d}t} = -\left((\mathrm{ka02}_1\,\cdot\,[\mathrm{PC/CRP}]\,\cdot\,[\mathrm{C1}]\,-\,\mathrm{ka02}_2\,\cdot\,[\mathrm{PC/CRP/C1}])\right) - \left((\mathrm{ke02}_1\,\cdot\,[\mathrm{GlcNac/LF/CRP}]\,\cdot\,[\mathrm{C1}]\,-\,\mathrm{ke02}_2\,\cdot\,[\mathrm{GlcNac/LF/CRP/C1}])\right) - \left((\mathrm{kd05}_1\,\cdot\,[\mathrm{PC/CRP/LF}]\,\cdot\,[\mathrm{C1}]\,-\,\mathrm{kd05}_2\,\cdot\,[\mathrm{PC/CRP/LF/C1}])\right) - \left((\mathrm{kd09}_1\,\cdot\,[\mathrm{PC/CRP/LF/MASP}]\,\cdot\,[\mathrm{C1}]\,-\,\mathrm{kd09}_2\,\cdot\,[\mathrm{PC/CRP/LF/C1/MASP}])\right)$$

$$\frac{\mathrm{d}\,([\mathrm{PC/CRP/C1}]\,)}{\mathrm{d}t} = +\left((\mathrm{ka02}_1\,\cdot\,[\mathrm{PC/CRP}]\,\cdot\,[\mathrm{C1}]\,-\,\mathrm{ka02}_2\,\cdot\,[\mathrm{PC/CRP/C1}])\right)$$

$$\frac{\mathrm{d}\,([\mathrm{C2a}]\,)}{\mathrm{d}t} = +\left(\frac{\mathrm{kd04}_1\,\cdot\,[\mathrm{PC/CRP/LF/MASP}]\,\cdot\,[\mathrm{C2}]}{\mathrm{kd04}_2\,+\,[\mathrm{C2}]}\right) + \left(\frac{\mathrm{kb04}_1\,\cdot\,[\mathrm{GlcNac/LF/MASP}]\,\cdot\,[\mathrm{C2}]}{\mathrm{kb04}_2\,+\,[\mathrm{C2}]}\right) + \left(\frac{\mathrm{ke04}_1\,\cdot\,[\mathrm{GlcNac/LF/CRP/C1}]\,\cdot\,[\mathrm{C2}]}{\mathrm{ke04}_2\,+\,[\mathrm{C2}]}\right) + \left(\mathrm{kf05}\,\cdot\,[\mathrm{C4b/C2a}]\,\cdot\,[\mathrm{C4BP}]\right) + \left(\frac{\mathrm{ka04}_1\,\cdot\,[\mathrm{PC/CRP/C1}]\,\cdot\,[\mathrm{C2}]}{\mathrm{ka04}_2\,+\,[\mathrm{C2}]}\right) + \left(\frac{\mathrm{kd07}_1\,\cdot\,[\mathrm{PC/CRP/LF/C1}]\,\cdot\,[\mathrm{C2}]}{\mathrm{kd07}_2\,+\,[\mathrm{C2}]}\right) + \left(\frac{\mathrm{ke07}_1\,\cdot\,[\mathrm{GlcNac/LF/CRP/MASP}]\,\cdot\,[\mathrm{C2}]}{\mathrm{ke07}_2\,+\,[\mathrm{C2}]}\right) - \left((\mathrm{kc01}_1\,\cdot\,[\mathrm{C4b}]\,\cdot\,[\mathrm{C2a}]\,-\,\mathrm{kc01}_2\,\cdot\,[\mathrm{C4b/C2a}])\right) + \left(\frac{\mathrm{kd11}_1\,\cdot\,[\mathrm{PC/CRP/LF/C1/MASP}]\,\cdot\,[\mathrm{C2}]}{\mathrm{kd11}_2\,+\,[\mathrm{C2}]}\right) + \left(\frac{\mathrm{kg04}_1\,\cdot\,[\mathrm{GlcNac/HF/MASP}]\,\cdot\,[\mathrm{C2}]}{\mathrm{kg04}_2\,+\,[\mathrm{C2}]}\right)$$

$$\frac{\mathrm{d}\,([\mathrm{C2b}]\,)}{\mathrm{d}t} = +\left(\frac{\mathrm{kd04}_1\,\cdot\,[\mathrm{PC/CRP/LF/MASP}]\,\cdot\,[\mathrm{C2}]}{\mathrm{kd04}_2\,+\,[\mathrm{C2}]}\right) + \left(\frac{\mathrm{kb04}_1\,\cdot\,[\mathrm{GlcNac/LF/MASP}]\,\cdot\,[\mathrm{C2}]}{\mathrm{kb04}_2\,+\,[\mathrm{C2}]}\right) + \left(\frac{\mathrm{ke04}_1\,\cdot\,[\mathrm{GlcNac/LF/CRP/C1}]\,\cdot\,[\mathrm{C2}]}{\mathrm{ke04}_2\,+\,[\mathrm{C2}]}\right) + \left(\frac{\mathrm{ka04}_1\,\cdot\,[\mathrm{PC/CRP/C1}]\,\cdot\,[\mathrm{C2}]}{\mathrm{ka04}_2\,+\,[\mathrm{C2}]}\right) + \left(\frac{\mathrm{kd07}_1\,\cdot\,[\mathrm{PC/CRP/LF/C1}]\,\cdot\,[\mathrm{C2}]}{\mathrm{kd07}_2\,+\,[\mathrm{C2}]}\right) + \left(\frac{\mathrm{ke07}_1\,\cdot\,[\mathrm{GlcNac/LF/CRP/MASP}]\,\cdot\,[\mathrm{C2}]}{\mathrm{ke07}_2\,+\,[\mathrm{C2}]}\right) + \left(\frac{\mathrm{kd11}_1\,\cdot\,[\mathrm{PC/CRP/LF/C1/MASP}]\,\cdot\,[\mathrm{C2}]}{\mathrm{kd11}_2\,+\,[\mathrm{C2}]}\right) + \left(\frac{\mathrm{kg04}_1\,\cdot\,[\mathrm{GlcNac/HF/MASP}]\,\cdot\,[\mathrm{C2}]}{\mathrm{kg04}_2\,+\,[\mathrm{C2}]}\right)$$

$$
\begin{aligned}
\frac{\mathrm{d}\left([\mathrm{C4b/C2a}]\right)}{\mathrm{d}t} = &- \left(\mathrm{kf03} \cdot [\mathrm{C4b/C2a}] \cdot [\mathrm{C4BP}]\right) \\
&- \left(\mathrm{kf05} \cdot [\mathrm{C4b/C2a}] \cdot [\mathrm{C4BP}]\right) \\
&- \left(\left(\mathrm{kf06_1} \cdot [\mathrm{C4b/C2a}] \cdot [\mathrm{C4BP}] - \mathrm{kf06_2} \cdot [\mathrm{C4b/C2a/C4BP}]\right)\right) \\
&- \left(\mathrm{k1_{(tmp3)}} \cdot [\mathrm{C4b/C2a}]\right) \\
&+ \left(\left(\mathrm{kc01_1} \cdot [\mathrm{C4b}] \cdot [\mathrm{C2a}] - \mathrm{kc01_2} \cdot [\mathrm{C4b/C2a}]\right)\right) \\
&- \left(\left(\mathrm{kc04_1} \cdot [\mathrm{C4b/C2a}] - \mathrm{kc04_2} \cdot [\mathrm{dC4b/C2a}]\right)\right)
\end{aligned}
$$

$$
\frac{\mathrm{d}\left([\mathrm{C3}]\right)}{\mathrm{d}t} = - \left(\mathrm{kc02} \cdot [\mathrm{C4b/C2a}] \cdot [\mathrm{C3}]\right)
$$

$$
\frac{\mathrm{d}\left([\mathrm{C3a}]\right)}{\mathrm{d}t} = + \left(\mathrm{kc02} \cdot [\mathrm{C4b/C2a}] \cdot [\mathrm{C3}]\right)
$$

$$
\begin{aligned}
\frac{\mathrm{d}\left([\mathrm{C3b}]\right)}{\mathrm{d}t} = &- \left(\mathrm{k1_{(tmp2)}} \cdot [\mathrm{C3b}]\right) \\
&+ \left(\mathrm{kc02} \cdot [\mathrm{C4b/C2a}] \cdot [\mathrm{C3}]\right) \\
&- \left(\left(\mathrm{kc03_1} \cdot [\mathrm{C3b}] - \mathrm{kc03_2} \cdot [\mathrm{dC3b}]\right)\right)
\end{aligned}
$$

$$
\frac{\mathrm{d}\left([\mathrm{dC3b}]\right)}{\mathrm{d}t} = + \left(\left(\mathrm{kc03_1} \cdot [\mathrm{C3b}] - \mathrm{kc03_2} \cdot [\mathrm{dC3b}]\right)\right)
$$

$$
\begin{aligned}
\frac{\mathrm{d}\left([\mathrm{MASP}]\right)}{\mathrm{d}t} = &- \left(\left(\mathrm{kb02_1} \cdot [\mathrm{GlcNac/LF}] \cdot [\mathrm{MASP}] - \mathrm{kb02_2} \cdot [\mathrm{GlcNac/LF/MASP}]\right)\right) \\
&- \left(\left(\mathrm{ke05_1} \cdot [\mathrm{GlcNac/LF/CRP}] \cdot [\mathrm{MASP}] - \mathrm{ke05_2} \cdot [\mathrm{GlcNac/LF/CRP/MASP}]\right)\right) \\
&- \left(\left(\mathrm{kd08_1} \cdot [\mathrm{PC/CRP/LF/C1}] \cdot [\mathrm{MASP}] - \mathrm{kd08_2} \cdot [\mathrm{PC/CRP/LF/C1/MASP}]\right)\right) \\
&- \left(\left(\mathrm{kg02_1} \cdot [\mathrm{GlcNac/HF}] \cdot [\mathrm{MASP}] - \mathrm{kg02_2} \cdot [\mathrm{GlcNac/HF/MASP}]\right)\right) \\
&- \left(\left(\mathrm{kd02_1} \cdot [\mathrm{PC/CRP/LF}] \cdot [\mathrm{MASP}] - \mathrm{kd02_2} \cdot [\mathrm{PC/CRP/LF/MASP}]\right)\right)
\end{aligned}
$$

$$
\begin{aligned}
\frac{\mathrm{d}\left([\mathrm{dC4b/C2a}]\right)}{\mathrm{d}t} = &- \left(\left(\mathrm{kf07_1} \cdot [\mathrm{dC4b/C2a}] \cdot [\mathrm{C4BP}] - \mathrm{kf07_2} \cdot [\mathrm{dC4b/C2a/C4BP}]\right)\right) \\
&+ \left(\left(\mathrm{kc04_1} \cdot [\mathrm{C4b/C2a}] - \mathrm{kc04_2} \cdot [\mathrm{dC4b/C2a}]\right)\right)
\end{aligned}
$$

$$
\frac{\mathrm{d}\left([\mathrm{GlcNac}]\right)}{\mathrm{d}t} = - \left(\left(\mathrm{kb01_1} \cdot [\mathrm{GlcNac}] \cdot [\mathrm{LF}] - \mathrm{kb01_2} \cdot [\mathrm{GlcNac/LF}]\right)\right)
$$

$$
\begin{aligned}
\frac{\mathrm{d}\left([\mathrm{GlcNac/LF}]\right)}{\mathrm{d}t} = &+ \left(\left(\mathrm{kb01_1} \cdot [\mathrm{GlcNac}] \cdot [\mathrm{LF}] - \mathrm{kb01_2} \cdot [\mathrm{GlcNac/LF}]\right)\right) \\
&- \left(\left(\mathrm{kb02_1} \cdot [\mathrm{GlcNac/LF}] \cdot [\mathrm{MASP}] - \mathrm{kb02_2} \cdot [\mathrm{GlcNac/LF/MASP}]\right)\right) \\
&- \left(\left(\mathrm{ke01_1} \cdot [\mathrm{GlcNac/LF}] \cdot [\mathrm{CRP}] - \mathrm{ke01_2} \cdot [\mathrm{GlcNac/LF/CRP}]\right)\right)
\end{aligned}
$$

$$
\begin{aligned}
\frac{\mathrm{d}\left([\mathrm{LF}]\right)}{\mathrm{d}t} = &- \left(\left(\mathrm{kb01_1} \cdot [\mathrm{GlcNac}] \cdot [\mathrm{LF}] - \mathrm{kb01_2} \cdot [\mathrm{GlcNac/LF}]\right)\right) \\
&- \left(\left(\mathrm{kd01_1} \cdot [\mathrm{PC/CRP}] \cdot [\mathrm{LF}] - \mathrm{kd01_2} \cdot [\mathrm{PC/CRP/LF}]\right)\right)
\end{aligned}
$$

$$
\frac{\mathrm{d}\left([\mathrm{GlcNac/LF/MASP}]\right)}{\mathrm{d}t} = + \left(\left(\mathrm{kb02_1} \cdot [\mathrm{GlcNac/LF}] \cdot [\mathrm{MASP}] - \mathrm{kb02_2} \cdot [\mathrm{GlcNac/LF/MASP}]\right)\right)
$$

$$
\begin{aligned}
\frac{\mathrm{d}\left([\mathrm{PC/CRP/LF}]\right)}{\mathrm{d}t} = &- \left(\left(\mathrm{kd05_1} \cdot [\mathrm{PC/CRP/LF}] \cdot [\mathrm{C1}] - \mathrm{kd05_2} \cdot [\mathrm{PC/CRP/LF/C1}]\right)\right) \\
&- \left(\left(\mathrm{k1_{(tmp_f1)}} \cdot [\mathrm{C4BP}] \cdot [\mathrm{PC/CRP/LF}] - \mathrm{k2_{(tmp_f1)}} \cdot [\mathrm{C4BP/PC/CRP/LF}]\right)\right) \\
&+ \left(\left(\mathrm{kd01_1} \cdot [\mathrm{PC/CRP}] \cdot [\mathrm{LF}] - \mathrm{kd01_2} \cdot [\mathrm{PC/CRP/LF}]\right)\right) \\
&- \left(\left(\mathrm{kd02_1} \cdot [\mathrm{PC/CRP/LF}] \cdot [\mathrm{MASP}] - \mathrm{kd02_2} \cdot [\mathrm{PC/CRP/LF/MASP}]\right)\right)
\end{aligned}
$$

$$
\begin{aligned}
\frac{\mathrm{d}\left([\mathrm{PC/CRP/LF/MASP}]\right)}{\mathrm{d}t} = &- \left(\left(\mathrm{kd09_1} \cdot [\mathrm{PC/CRP/LF/MASP}] \cdot [\mathrm{C1}] - \mathrm{kd09_2} \cdot [\mathrm{PC/CRP/LF/C1/MASP}]\right)\right) \\
&+ \left(\left(\mathrm{kd02_1} \cdot [\mathrm{PC/CRP/LF}] \cdot [\mathrm{MASP}] - \mathrm{kd02_2} \cdot [\mathrm{PC/CRP/LF/MASP}]\right)\right)
\end{aligned}
$$

$$
\begin{aligned}
\frac{\mathrm{d}\left([\mathrm{GlcNac/LF/CRP}]\right)}{\mathrm{d}t} = &+ \left(\left(\mathrm{ke01_1} \cdot [\mathrm{GlcNac/LF}] \cdot [\mathrm{CRP}] - \mathrm{ke01_2} \cdot [\mathrm{GlcNac/LF/CRP}]\right)\right) \\
&- \left(\left(\mathrm{ke02_1} \cdot [\mathrm{GlcNac/LF/CRP}] \cdot [\mathrm{C1}] - \mathrm{ke02_2} \cdot [\mathrm{GlcNac/LF/CRP/C1}]\right)\right) \\
&- \left(\left(\mathrm{kf02_1} \cdot [\mathrm{C4BP}] \cdot [\mathrm{GlcNac/LF/CRP}] - \mathrm{kf02_2} \cdot [\mathrm{C4BP/GlcNac/LF/CRP}]\right)\right) \\
&- \left(\left(\mathrm{ke05_1} \cdot [\mathrm{GlcNac/LF/CRP}] \cdot [\mathrm{MASP}] - \mathrm{ke05_2} \cdot [\mathrm{GlcNac/LF/CRP/MASP}]\right)\right)
\end{aligned}
$$

$$
\frac{\mathrm{d}\left([\mathrm{GlcNac/LF/CRP/C1}]\right)}{\mathrm{d}t} = + \left(\left(\mathrm{ke02_1} \cdot [\mathrm{GlcNac/LF/CRP}] \cdot [\mathrm{C1}] - \mathrm{ke02_2} \cdot [\mathrm{GlcNac/LF/CRP/C1}]\right)\right)
$$

$$
\begin{aligned}
\frac{\mathrm{d}\left([\mathrm{C4BP}]\right)}{\mathrm{d}t} = &- \left(\left(\mathrm{kf01_1} \cdot [\mathrm{C4BP}] \cdot [\mathrm{PC/CRP}] - \mathrm{kf01_2} \cdot [\mathrm{C4BP/PC/CRP}]\right)\right) \\
&- \left(\left(\mathrm{kf02_1} \cdot [\mathrm{C4BP}] \cdot [\mathrm{GlcNac/LF/CRP}] - \mathrm{kf02_2} \cdot [\mathrm{C4BP/GlcNac/LF/CRP}]\right)\right) \\
&- \left(\left(\mathrm{kf04_1} \cdot [\mathrm{C4BP}] \cdot [\mathrm{C4b}] - \mathrm{kf04_2} \cdot [\mathrm{C4BP/C4b}]\right)\right) \\
&- \left(\left(\mathrm{kf06_1} \cdot [\mathrm{C4b/C2a}] \cdot [\mathrm{C4BP}] - \mathrm{kf06_2} \cdot [\mathrm{C4b/C2a/C4BP}]\right)\right) \\
&- \left(\left(\mathrm{kf07_1} \cdot [\mathrm{dC4b/C2a}] \cdot [\mathrm{C4BP}] - \mathrm{kf07_2} \cdot [\mathrm{dC4b/C2a/C4BP}]\right)\right) \\
&- \left(\mathrm{k1_{(tmp1)}} \cdot [\mathrm{C4BP}]\right) \\
&- \left(\left(\mathrm{k1_{(tmp_f1)}} \cdot [\mathrm{C4BP}] \cdot [\mathrm{PC/CRP/LF}] - \mathrm{k2_{(tmp_f1)}} \cdot [\mathrm{C4BP/PC/CRP/LF}]\right)\right)
\end{aligned}
$$

$$
\begin{aligned}
\frac{\mathrm{d}\left([\mathrm{C4BP/PC/CRP}]\right)}{\mathrm{d}t} &= +\left((\mathrm{kf01}_1 \cdot [\mathrm{C4BP}] \cdot [\mathrm{PC/CRP}] - \mathrm{kf01}_2 \cdot [\mathrm{C4BP/PC/CRP}])\right) \\
\frac{\mathrm{d}\left([\mathrm{C4BP/GlcNac/LF/CRP}]\right)}{\mathrm{d}t} &= +\left((\mathrm{kf02}_1 \cdot [\mathrm{C4BP}] \cdot [\mathrm{GlcNac/LF/CRP}] - \mathrm{kf02}_2 \cdot [\mathrm{C4BP/GlcNac/LF/CRP}])\right) \\
\frac{\mathrm{d}\left([\mathrm{iC4b/C2a}]\right)}{\mathrm{d}t} &= +\left(\mathrm{kf03} \cdot [\mathrm{C4b/C2a}] \cdot [\mathrm{C4BP}]\right) \\
\frac{\mathrm{d}\left([\mathrm{C4BP/C4b}]\right)}{\mathrm{d}t} &= +\left((\mathrm{kf04}_1 \cdot [\mathrm{C4BP}] \cdot [\mathrm{C4b}] - \mathrm{kf04}_2 \cdot [\mathrm{C4BP/C4b}])\right) \\
\frac{\mathrm{d}\left([\mathrm{C4b/C2a/C4BP}]\right)}{\mathrm{d}t} &= +\left((\mathrm{kf06}_1 \cdot [\mathrm{C4b/C2a}] \cdot [\mathrm{C4BP}] - \mathrm{kf06}_2 \cdot [\mathrm{C4b/C2a/C4BP}])\right) \\
\frac{\mathrm{d}\left([\mathrm{dC4b/C2a/C4BP}]\right)}{\mathrm{d}t} &= +\left((\mathrm{kf07}_1 \cdot [\mathrm{dC4b/C2a}] \cdot [\mathrm{C4BP}] - \mathrm{kf07}_2 \cdot [\mathrm{dC4b/C2a/C4BP}])\right) \\
\frac{\mathrm{d}\left([\mathrm{PC/CRP/LF/C1}]\right)}{\mathrm{d}t} &= +\left((\mathrm{kd05}_1 \cdot [\mathrm{PC/CRP/LF}] \cdot [\mathrm{C1}] - \mathrm{kd05}_2 \cdot [\mathrm{PC/CRP/LF/C1}])\right) \\
&\quad -\left((\mathrm{kd08}_1 \cdot [\mathrm{PC/CRP/LF/C1}] \cdot [\mathrm{MASP}] - \mathrm{kd08}_2 \cdot [\mathrm{PC/CRP/LF/C1/MASP}])\right) \\
\frac{\mathrm{d}\left([\mathrm{C4BP/PC/CRP/LF}]\right)}{\mathrm{d}t} &= +\left(\left(\mathrm{k1}_{(\mathrm{tmp_f 1})} \cdot [\mathrm{C4BP}] \cdot [\mathrm{PC/CRP/LF}] - \mathrm{k2}_{(\mathrm{tmp_f 1})} \cdot [\mathrm{C4BP/PC/CRP/LF}]\right)\right) \\
\frac{\mathrm{d}\left([\mathrm{GlcNac/LF/CRP/MASP}]\right)}{\mathrm{d}t} &= +\left((\mathrm{ke05}_1 \cdot [\mathrm{GlcNac/LF/CRP}] \cdot [\mathrm{MASP}] - \mathrm{ke05}_2 \cdot [\mathrm{GlcNac/LF/CRP/MASP}])\right) \\
\frac{\mathrm{d}\left([\mathrm{PC/CRP/LF/C1/MASP}]\right)}{\mathrm{d}t} &= +\left((\mathrm{kd08}_1 \cdot [\mathrm{PC/CRP/LF/C1}] \cdot [\mathrm{MASP}] - \mathrm{kd08}_2 \cdot [\mathrm{PC/CRP/LF/C1/MASP}])\right) \\
&\quad +\left((\mathrm{kd09}_1 \cdot [\mathrm{PC/CRP/LF/MASP}] \cdot [\mathrm{C1}] - \mathrm{kd09}_2 \cdot [\mathrm{PC/CRP/LF/C1/MASP}])\right) \\
\frac{\mathrm{d}\left([\mathrm{GlcNac/HF}]\right)}{\mathrm{d}t} &= +\left((\mathrm{kg01}_1 \cdot [\mathrm{X}] \cdot [\mathrm{HF}] - \mathrm{kg01}_2 \cdot [\mathrm{GlcNac/HF}])\right) \\
&\quad -\left((\mathrm{kg02}_1 \cdot [\mathrm{GlcNac/HF}] \cdot [\mathrm{MASP}] - \mathrm{kg02}_2 \cdot [\mathrm{GlcNac/HF/MASP}])\right) \\
\frac{\mathrm{d}\left([\mathrm{HF}]\right)}{\mathrm{d}t} &= -\left((\mathrm{kg01}_1 \cdot [\mathrm{X}] \cdot [\mathrm{HF}] - \mathrm{kg01}_2 \cdot [\mathrm{GlcNac/HF}])\right) \\
\frac{\mathrm{d}\left([\mathrm{GlcNac/HF/MASP}]\right)}{\mathrm{d}t} &= +\left((\mathrm{kg02}_1 \cdot [\mathrm{GlcNac/HF}] \cdot [\mathrm{MASP}] - \mathrm{kg02}_2 \cdot [\mathrm{GlcNac/HF/MASP}])\right) \\
\frac{\mathrm{d}\left([\mathrm{X}]\right)}{\mathrm{d}t} &= -\left((\mathrm{kg01}_1 \cdot [\mathrm{X}] \cdot [\mathrm{HF}] - \mathrm{kg01}_2 \cdot [\mathrm{GlcNac/HF}])\right)
\end{aligned}
$$
